# Myalgic Encephalomyelitis/Chronic Fatigue Syndrome: the biology of a neglected disease

**DOI:** 10.3389/fimmu.2024.1386607

**Published:** 2024-06-03

**Authors:** Hayley E. Arron, Benjamin D. Marsh, Douglas B. Kell, M. Asad Khan, Beate R. Jaeger, Etheresia Pretorius

**Affiliations:** ^1^ Department of Physiological Sciences, Faculty of Science, Stellenbosch University, Stellenbosch, South Africa; ^2^ MRCPCH Consultant Paediatric Neurodisability, Exeter, Devon, United Kingdom; ^3^ Department of Biochemistry and Systems Biology, Institute of Systems, Molecular and Integrative Biology, Faculty of Health and Life Sciences, University of Liverpool, Liverpool, United Kingdom; ^4^ The Novo Nordisk Foundation Centre for Biosustainability, Technical University of Denmark, Lyngby, Denmark; ^5^ Directorate of Respiratory Medicine, Manchester University Hospitals, Wythenshawe Hospital, Manchester, United Kingdom; ^6^ Long COVID department, Clinic St Georg, Bad Aibling, Germany

**Keywords:** myalgic encephalomyelitis/chronic fatigue syndrome, pathology, treatment, pathophysiology, diagnostic criteria

## Abstract

Myalgic Encephalomyelitis/Chronic Fatigue Syndrome (ME/CFS) is a chronic, debilitating disease characterised by a wide range of symptoms that severely impact all aspects of life. Despite its significant prevalence, ME/CFS remains one of the most understudied and misunderstood conditions in modern medicine. ME/CFS lacks standardised diagnostic criteria owing to variations in both inclusion and exclusion criteria across different diagnostic guidelines, and furthermore, there are currently no effective treatments available. Moving beyond the traditional fragmented perspectives that have limited our understanding and management of the disease, our analysis of current information on ME/CFS represents a significant paradigm shift by synthesising the disease’s multifactorial origins into a cohesive model. We discuss how ME/CFS emerges from an intricate web of genetic vulnerabilities and environmental triggers, notably viral infections, leading to a complex series of pathological responses including immune dysregulation, chronic inflammation, gut dysbiosis, and metabolic disturbances. This comprehensive model not only advances our understanding of ME/CFS’s pathophysiology but also opens new avenues for research and potential therapeutic strategies. By integrating these disparate elements, our work emphasises the necessity of a holistic approach to diagnosing, researching, and treating ME/CFS, urging the scientific community to reconsider the disease’s complexity and the multifaceted approach required for its study and management.

## Introduction

Myalgic Encephalomyelitis/Chronic Fatigue Syndrome (ME/CFS) or Systemic Exertion Intolerance Disease (SEID) (1–3) (hereafter referred to as ME/CFS) is a debilitating chronic multisystem illness. ME/CFS is estimated to have a global prevalence ranging from 0.1-0.8% (4–7). It is thought to affect some 17 to 24 million people worldwide (8) and the United States (U.S.) Centres for Disease Control and Prevention (CDC) and the U.S. National Academy of Medicine estimate that there are approximately 836,000 to 2.5 million (2, 9) individuals diagnosed with ME/CFS in the U.S., with a quarter of these patients thought to be housebound or bedbound (10). Additionally, current data from the United Kingdom (U.K.) Biobank has indicated that there are more than 250,000 individuals suffering from ME/CFS in England and Wales (11), with a prevalence of 0.2% in three regions of England (6).

ME/CFS impacts all ages, races, and socioeconomic groups (4, 12). Most patients tend to be diagnosed around middle age (13), but diagnosis has been made in individuals as young as three years old and as old as 77 years (2, 12). Black and Hispanic populations appear to have a higher prevalence of ME/CFS with worse severity than other racial groups (9, 14). Women are affected 2-3 times more frequently than men (2, 4, 8, 11, 12).

Due to the disabling symptoms which include cognitive and physical impairment worsened by exertion (15–17), there is a significant economic burden created by ME/CFS (4) as many patients are unemployed and less than a fifth work full-time (11). It is thought that up to 75% of ME/CFS patients are unable to work (10). ME/CFS is thought to cost the U.S. between $18 and $24 billion annually (18), and £3.3 billion annually in the U.K (19).

**Figure 1:**
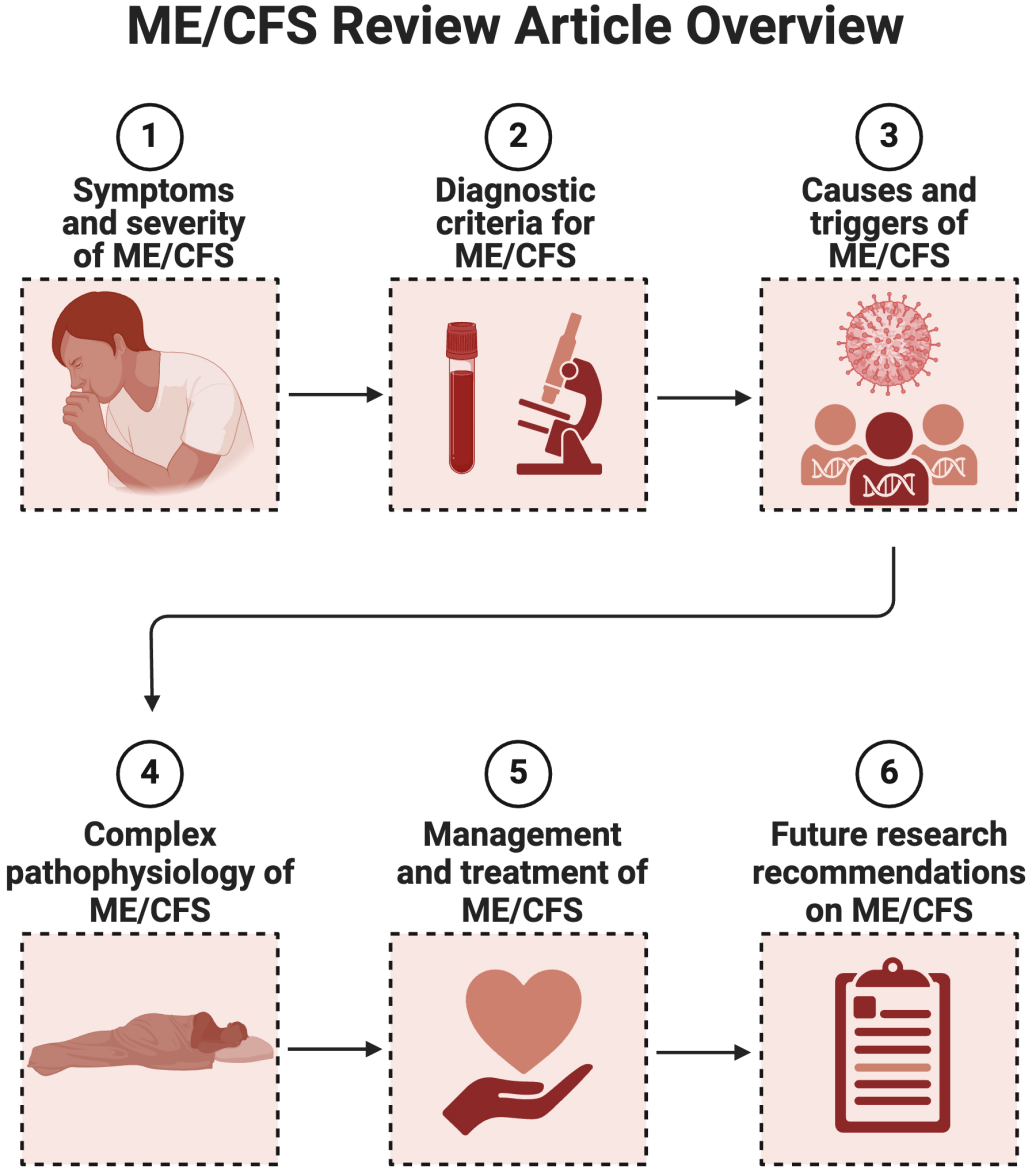
Overview review article of ME/CFS as a complex and multifactorial condition (Created with Biorender.com).

In this review paper, we will focus on describing ME/CFS in terms of symptoms, severity and burden, diagnostic criteria, causes and triggers; followed by an overview of the complex pathophysiology and management of the condition. We will conclude by listing research priorities for the future. See **Figure 1** for a content overview.

### ME/CFS symptoms

**Figure 2:**
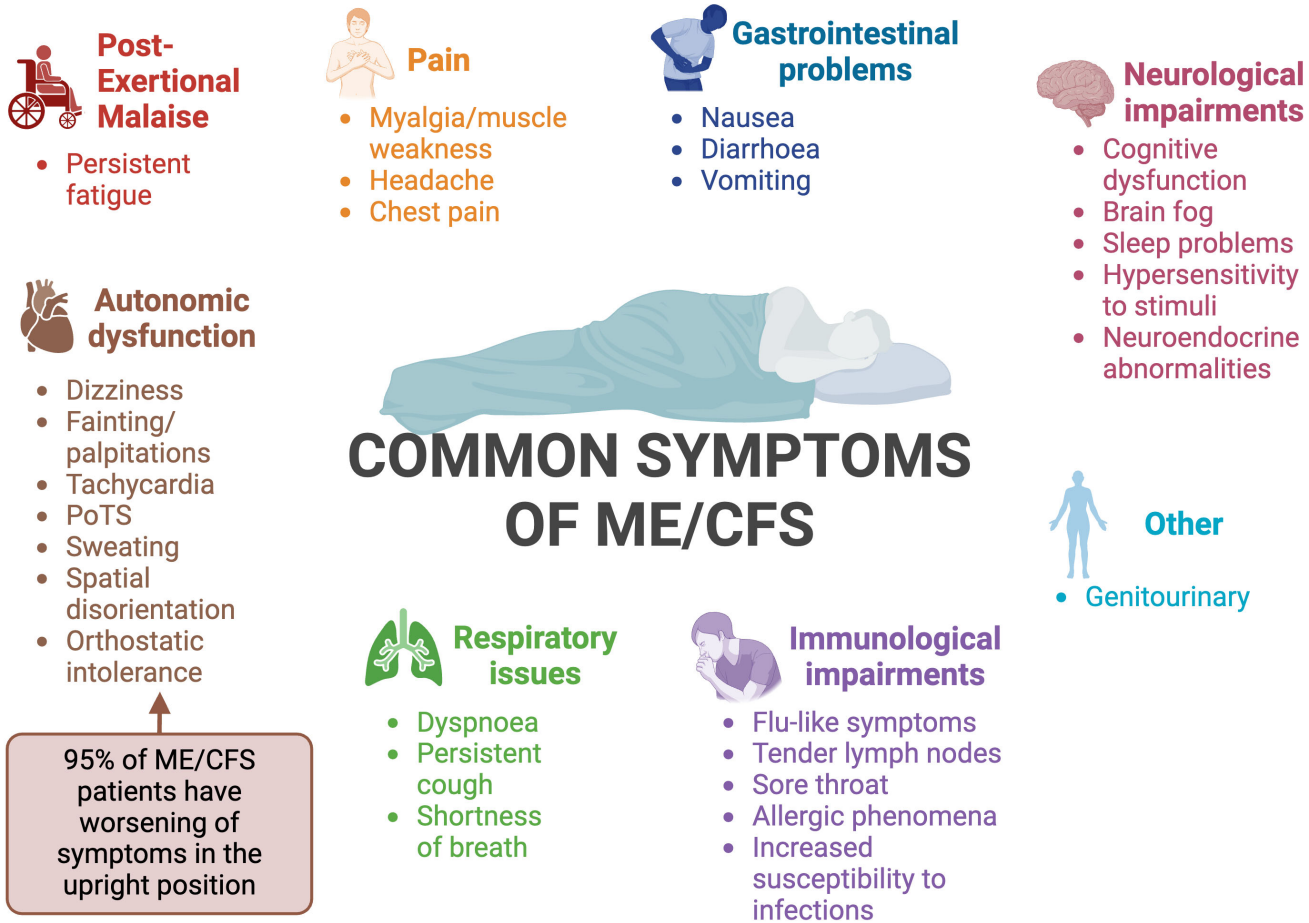
Summary of the common symptoms of ME/CFS (12, 15, 22–26). PoTS, postural orthostatic tachycardia syndrome. Created with Biorender.com.

ME/CFS is a heterogeneous disease (16, 20), meaning that patients display varying symptoms and several body systems are impacted (11). Although many of the symptoms of ME/CFS overlap with other diseases, one feature that sets ME/CFS apart is a worsening of symptoms in response to relatively minor physical, cognitive, orthostatic or even emotional exertion (13). This phenomenon is known as post-exertional malaise (PEM) or PESE (post exertional symptom exacerbation). Following exertion, patients experience reduced mental and physical stamina, accompanied by accelerated muscle and cognitive fatigue (15–17). PEM is characterised by a delayed onset, prolonged duration, and an intensity disproportionate to the precipitating exertion (15, 16). Fatigue is a prominent feature in most patients; in contrast to physiological tiredness, fatigue is not alleviated regardless of how much patients sleep or rest (2, 21). Common symptoms of ME/CFS are summarised in **Figure 2**.

### ME/CFS severity classifications

The long-term outlook for ME/CFS patients varies significantly (11). An important prognostic factor is how the illness is managed in its initial stages (27). The chance of full recovery has been estimated to be only 5% (28) as most patients do not regain their full pre-ME/CFS function; a third of patients deteriorate to being house or bedbound (8). Hence, ME/CFS significantly reduces a patient’s quality of life (11, 24, 29–31), impacting activities of daily living, socialising, family life, emotional wellbeing, work, and education (11, 23). Numerous researchers have found that individuals suffering from ME/CFS have a lower quality of life than people with other chronic illnesses, including multiple sclerosis (MS) (32), lung disease (33), rheumatoid arthritis (RA) (33–35), and some forms of cancer (11, 33, 35). Similar to other disabling chronic illnesses, resultant depression and anxiety in ME/CFS are frequent (36) and there is a higher risk of suicide in ME/CFS (37).

Patients’ symptoms can usually be categorised into four levels of severity; mild, moderate, severe, and very severe (11, 29, 38). In mild severity ME/CFS, an individual is still able to attend work or education and perform light tasks (11). However, they are likely to experience difficulties with mobility, and their social life is impacted. In moderate ME/CFS, individuals experience impaired mobility, meaning they are likely unable to attend work or education, cannot undertake activities alone, have decreased sleep quality, and need frequent rest. People with severe ME/CFS are usually housebound (11) and have a functional capacity ranging from 5% to 15% of normal functioning (39). They also experience cognitive difficulties and are hypersensitive to external stimuli (11). Lastly, people with very severe ME/CFS are bedbound (11) and have a functional capacity of less than 5% (39). These individuals are usually completely dependent on others for care, require tube feeding, are unable to complete personal hygiene, and are highly sensitive to sensory stimuli (11). ME/CFS has been described as a fluctuating condition as symptoms change in nature and severity sporadically (40). 61% of patients have reported being bedbound on their worst days (41) and at least one-quarter of ME/CFS patients have recounted being housebound or bedbound at some point in their lives (2). Hence, patients must adapt to life with regular flare-ups and relapses (11, 42).

In one survey of 1418 ME/CFS patients (43), it was found that 98.5% (n=1397) of the ME/CFS participants struggled with performing daily tasks and more than half (n=775) were completely unable to complete their usual activities. Additionally, 93.9% (n=1331) of participants experienced moderate (n=976) or extreme (n=355) pain, 88.6% (n=1256) of patients had trouble with mobility, and 67.3% (n=954) experienced trouble with washing or dressing themselves. However, the severity of ME/CFS means that family members or caretakers are also severely impacted. Of 1418 respondent caretakers/family members, 96.1% (n=1362) felt worried in relation to their family member’s ME/CFS, 84.7% (n=1201) found it difficult to care for their family members suffering from ME/CFS, and 91.8% (n=1302) reported that their family activities were affected. Additionally, caring for a member with ME/CFS negatively impacts sleep, work, holidays, finances, and sex life (43).

### Diagnostic criteria for ME/CFS

#### The original neurological classification of ME/CFS

In 1969, the World Health Organisation (WHO) classified ME/CFS as a neurological disease (44) based on the neurological features of the disease. Positron emission tomography (PET) brain imaging of ME/CFS patients also displays neuroinflammation (45–47). Additionally, abnormalities of the white (23, 48–50) and grey (23) matter have been noted. This neuroinflammation is likely to correlate with cognitive impairments such as lowered information processing speed, decreased reaction time, slower working memory, and reduced attention (51).

#### Available diagnostic criteria

**Figure 3:**
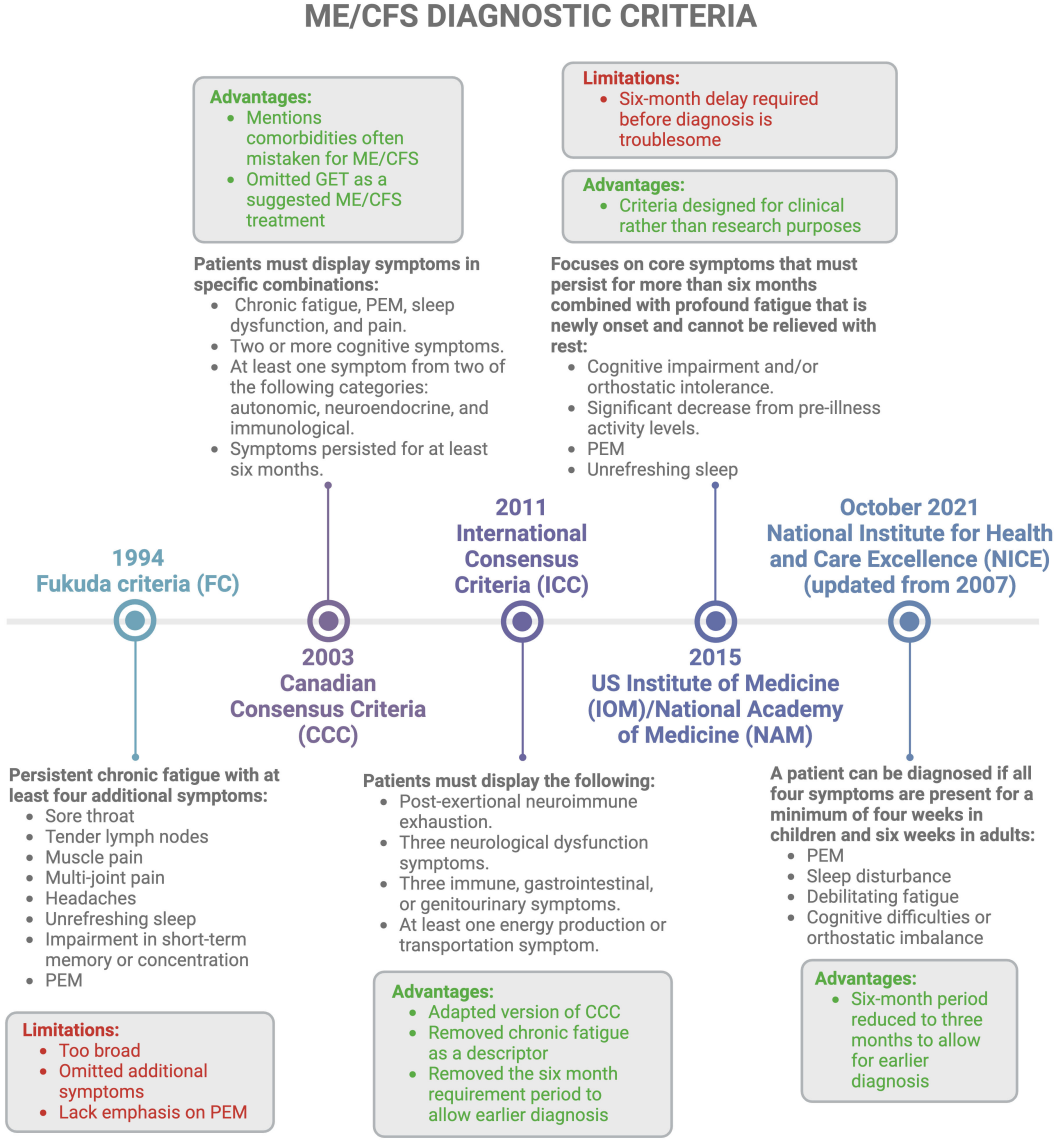
Different diagnostic criteria available for ME/CFS with a concise history of timelines (1, 2, 11, 15, 16, 22, 52–54). Created with Biorender.com. GET, graded exercise therapy; ME/CFS, Myalgic Encephalomyelitis/Chronic Fatigue Syndrome; PEM, post-exertional malaise.

Despite its high prevalence, there are still no universally accepted clinical criteria to characterise ME/CFS, making early and accurate diagnosis difficult (52). Different ME/CFS diagnostic criteria exist, as described in **Figure 3**. Additionally, many adult ME/CFS patients make use of the self-scoring Bell Chronic fatigue immune dysfunction syndrome (CFIDS) disability scale, a symptom scale created by Dr David Bell in 1995 (55). However, this classification is subjective, and scores may change due to the fluctuating nature of the disease; they may also vary depending on the physician doing the scoring. Another problem is that a patient may experience one score in one region, but a differing score in another region, making it difficult for the physician to make an overall decision. Despite the large number of criteria for the diagnosis of ME/CFS, it is still unclear which one is the most useful and validated. Current diagnostics rely on medical history, physical examination, and clinical observations, which often require many trips to the doctor and can be exhausting for a patient (11, 23, 56).

Additionally, PEM which can vary from very minimal to extremely severe (e.g. affecting speaking, thinking, eating and drinking, and orthostatic tolerance) is considered a key diagnostic feature of ME/CFS; this is paradoxical as severe and very severe patients are incapable of even the slightest exertion (39). Moreover, diagnosis relies heavily on symptom-based exclusion of other disorders (2, 11, 15, 16, 22, 56, 57), since ME/CFS symptoms overlap with many other medical conditions (23). In essence, since ME/CFS can be a “diagnosis of last resort” (1, 26), it can take years for individuals to receive a diagnosis (22). This prolonged diagnostic process is compounded by differences in exclusion criteria across various definitions of ME/CFS, as highlighted by Jason et al., 2012 (54). Hence, re-evaluation of these diagnostic criteria is essential (57).

## Probable predisposition and causes of ME/CFS

The diverse symptom presentation and patient heterogeneity indicate that ME/CFS is likely to have a multifactorial origin (23, 30, 53, 58), with multiple physiological processes implicated in the pathogenesis (52, 59). It is unlikely that one single protein or RNA transcript will be consistent across the disease spectrum (60). It has been proposed that stressful or traumatic incidents in genetically susceptible individuals may trigger the development of ME/CFS symptoms (58, 61). These stressors can include acute infections (61), emotional stress (62), and major life events (61). However, other stressors such as a quick walk, a glass of wine, or a temperature change have also been known to trigger relapses in ME/CFS (63). The next paragraphs will discuss genetic predisposition and susceptibility as an underlying risk factor for ME/CFS - the superimposition of external stressors and exposures (59) results in a state of “aberrant homeostasis”, where a temporary homeostatic state of functioning occurs, but functions at a less optimum level (64).

### Genetic predisposition

Unfortunately, there is little consensus among researchers on the genetic, cellular, and molecular influences that alter the risk of developing ME/CFS (21). However, it is thought that ME/CFS may have a genetic predisposition (26, 29, 56, 61, 65–68) as members of the same family have frequently been diagnosed with ME/CFS (65, 67, 68), though they will also tend to share similar lifestyles and cultures. Similarly, genealogic analyses and twin studies have shown a genetic link between ME/CFS patients and their offspring, ensuing a heightened risk of developing ME/CFS in the offspring (26, 66, 69). As expected (70), these diagnoses do not seem to follow predictable Mendelian patterns, suggesting that there are multiple genes and alleles that increase the risk of developing ME/CFS (56). Various studies have found that some ME/CFS patients identified by the Fukuda criteria have a significant excess relatedness for close (first or second degree) and distant (third degree) relatives (66, 68). A large review (56) noted that three studies estimated the narrow-sense heritability (h^2^) in large cohorts, and two of these studies reported non-zero results, suggesting a heritability risk for ME/CFS. However, other studies have shown no evidence of heritability. One analysis of the U.S. health insurance resulted in a high narrow-sense heritability (h^2 =^ 0.48) (71), while an analysis of U.K. Biobank self-reported ME/CFS patients approximated the h^2^ to be 0.08 with a low confidence (72). Furthermore, another large twin-based study produced inconclusive results (73).

Some polymorphisms in genes related to the immunomodulatory response have been identified (69, 74), such as an increase of TNF-857 TT and CT genotypes (p=0.002), and a significant decrease of IFN gamma low producers (A/A) (P=0.04) in ME/CFS patients in respect to controls (74). Polymorphisms impacting hormone action have also been noted (69, 75). For example, NR3C1-1F DNA methylation was found reduced in patients with ME/CFS and coincides with the hypothalamic pituitary-adrenal (HPA) axis hypofunction hypothesis (75). The metabolic kynurenic pathways is likely also implicated in ME/CFS, owing to common mutations in IDO2 (76) such as R248W and Y359STOP (77). However, these genetic associations are inconsistent as studies typically have quality-control issues (56). Additionally, the Gln27 mutation (a polymorphism in the B2dR genes) has been associated with adolescent ME/CFS (78). Such Gln27 mutations, along with Arg16 mutations in adults, can result in an unfavourable cardiovascular profile (79–81). On the other hand, genome-wide association studies (GWAS) studies performed on ME/CFS patient samples from the U.K. Biobank have found no DNA variant repeated in the multiple analyses (82). However, it is not entirely certain how many of these patients met ME/CFS criteria, suggesting that replication studies should be done in future to help us understand the genes, cellular processes, and tissues or cells that causally change the risk for developing ME/CFS. Additionally, pinpointing genetic risk factors for ME/CFS will help combat the disbelief some health professionals have towards ME/CFS (56).

There are also genetic predispositions that render certain individuals susceptible to developing autoimmune diseases (83) and even after Epstein-Barr virus (EBV) infection (30, 84), such as human leukocyte antigen (HLA) variants (30, 84, 85) that have experienced selective pressures whilst co-evolving with pathogens (86, 87). HLA proteins are crucial for the immune system as they distinguish foreign pathogens from self-cells (56). An increased frequency of HLA-DQA1 alleles and reduced expression of HLA-DRB1 was found associated with ME/CFS (88). Additionally, in patients diagnosed with the CCC, the HLA types HLA-C*07:04 or HLA-DQB1*03:03 were shown to be significantly linked to ME/CFS status when using the CCC (89). Since 10% of ME/CFS patients have these alleles, their risk is altered around 1.5-2.0-fold, meaning genetic differences in the immune system may change the risk of developing ME/CFS (89). Some haplotypes also appear to be less resistant to EBV infection, such as DR2-DQ6, DR3-DQ2, and DR4-DQ8, making them more likely to develop EBV-related disorders (90). Although, few researchers think that mitochondrial DNA variants can explain the prevalence of ME/CFS (91–93).

### Viral triggers and reactivation

**Figure 4:**
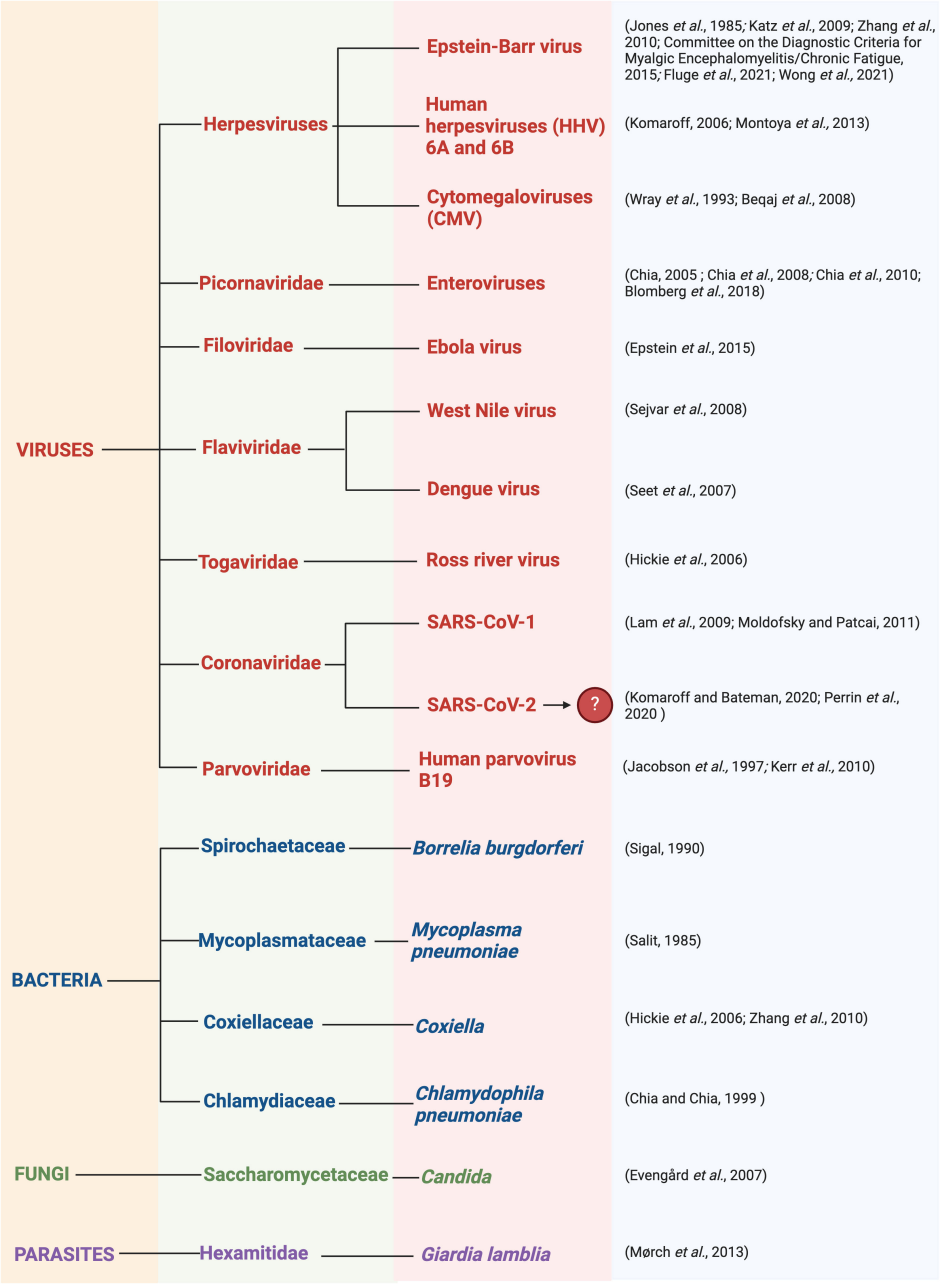
Infectious pathogens thought to promote the development of ME/CFS including viruses, bacteria, fungi, and parasites (2, 25, 29, 85, 102–126). Created with Biorender.com.

As in other chronic diseases, ME/CFS involves an asymptomatic predisposition stage, then a prodromal stage, followed by symptomatic disease (59). Infection (30, 61, 94–97) is often reported to be a common trigger for the development of ME/CFS, as many patients recognise that the onset of their symptoms coincided with an infectious episode (2, 58, 98). This is recounted by more than 80% of patients (98) and it has been estimated that two-thirds of ME/CFS cases arise following viral infection (85, 99). This is supported by the fact that several ME/CFS outbreaks have occurred in the same geographical region simultaneously (12). Clustering provides strong support for the involvement of infectious agents, as with some cases of MS that ‘appeared’ (100) in the Faroe Islands during World War II upon the arrival of foreign army corps, and was eventually shown to be due to EBV (101). Additionally, many ME/CFS patients experience symptoms similar to bacterial or viral infections which may correlate to specific regions. Infectious pathogens thought to promote the development of ME/CFS are summarised in **Figure 4**.

It is thought that many chronic illnesses, including ME/CFS, originate with reactivation of latent or dormant viruses that are present in the body, caused by a change in physiological conditions (127). During acute illness, direct consequences of viral reactivation or infection are thought to occur in localised areas such as the brain and neuronal cells, muscle cells, and cardiac myocytes [Extended Figure 8 in (127)]. Since these tissues are rich in mitochondria, it can result in neuronal and autonomic nervous system abnormalities, as well as immune modulation. However, with chronic illness, indirect consequences of viral reactivation or infection are proposed to occur. This occurs in peripheral circulation and can promote symptoms such as postural orthostatic tachycardia syndrome (PoTS), PEM, fatigue, endothelial cell dysfunction, platelet hyperactivation, mast cell activation, and connective tissue disorders. In this chronic phase, it is thought that auto recovery is difficult (127).

Viral pathogens that often persist in a dormant manner include herpesviruses such as the extensively studied EBV (52, 94, 95, 113). Reactivation of herpesvirus has interestingly been hypothesised as a key mechanism in the development of ME/CFS (128). In support of this theory, one paper investigated the possibility of EBV-encoded proteins existing in ME/CFS, such as BRRF1 and BLLF3, and the effects they may have (52). BRRF1 plays a role in the switch between latent and lytic EBV replication (129), while BLLF3 encodes a dUTPase and is expressed in the early phase of EBV lytic replication (52). Similarly, evidence for antibodies against HSV-1 (127), EBV (127, 130, 131), and HHV-6 (130) dUTPase proteins has been identified in ME/CFS. These dUTPase proteins, along with other viral dUTPases such as HSV-1 and HHV-6, are thought to promote cellular damage and promote autoimmune features, as well as promoting mitochondrial fusion and decreasing energy production (127). This antibody presence is thought to potentially elevate cytokine and chemokine levels, which is dependent on toll-like receptor (TLR)-2 signalling and causes NF-κB activation (132, 133). Hence, the EBV UTPase may act as a novel pathogen-associated molecular pattern (PAMP) ligand protein for TLR2 (132).

Although EBV proteins may play a role in the progression of ME/CFS (52), researchers have hypothesised that the virus has a third state termed the abortive/leaky/lytic replication, as has been suggested upon analysis of the EBV genome in T and NK cells of patients with chronic EBV infection (134–136). The presence of EBV dUTPases may cause neurological abnormalities in ME/CFS via changes in gene expression, resulting in modulated neurological circuits (137). Moreover, EBV dUTPases could also induce immune dysfunction (52).

However, earlier serological studies on EBV and ME/CFS using classical EBV antigens have resulted in contradicting results that illustrate no upregulation of EBV-encoded proteins (99, 138–147). More advanced studies using peptide microarray (148) and suspension multiplex immunoassay (149) have also not found a significantly higher EBV anti-immunoglobulin (Ig)-G response in ME/CFS patients in comparison to controls. Most studies have not reported a significant increase in the viral load in ME/CFS patients in comparison to controls. Due to this evidence, some researchers believe that herpesviruses as one of the causes of ME/CFS is a “fading” hypothesis (150). However, a trigger does not have to linger. Equally, heterogeneity in patient groups, the absence of uniformity in case definitions, and differences in reliability and precision could have resulted in there being no correlation between the viral load and serological data (61, 99, 103, 109).

That said, serological data are probably of limited use as an indicator of EBV reactivation (151). Although there is substantial evidence that ME/CFS has a viral trigger (94, 95), symptom severity and burden are often not related to the severity of the triggering viral infection and its symptomatology. Additionally, there are alternative theories differing to a post-viral causation, as noted later in the text.

### Toxin and drug exposure

Some also hypothesise that toxin exposure could trigger ME/CFS (61), such as organophosphate compounds (152, 153) and heavy metals (154). In the early 1960s, researchers found that workers with chronic exposure to organophosphates, mainly in insecticides and after sheep dipping, experienced persistent central nervous system (CNS) changes (153). This included disabling fatigue exacerbated by exercise and associated with myalgia, excessive sleep, night sweats, irritable bowel syndrome (IBS) symptoms, and mental changes. It was hypothesised that the organophosphates induce various abnormalities such as an elevated prevalence of lymphoproliferative disorders associated with impaired natural killer (NK) cell and cytotoxic T cell function. Additionally, exposure to heavy metals, such as cadmium, may also contribute to the development of ME/CFS (154). Cadmium is a widespread environmental and occupational heavy metal pollutant and can potentially cause neurological symptoms. Cadmium induces neuronal death in cortical neurons through a combination of apoptosis and necrosis, involving reactive oxygen species (ROS) generation and lipid peroxidation (155). This action may explain decreased grey matter volume in ME/CFS (156), as well as certain effects on the CNS such as reduced attention levels and memory (157). Exposure to cadmium also potentially reduces cerebral blood flow (158) [particularly cortical blood flow (159)], as cadmium has a disruptive effect on angiogenesis (160). This reduced blood flow can result in neurological dysfunction and the abnormal neuroimaging observed in ME/CFS (158). Cadmium may accentuate inflammatory processes, which may in turn disrupt the HPA axis and trigger symptoms of ME/CFS (161, 162). However, the exact organ that cadmium toxicity targets is still unclear (154).

Additionally, cases of ME/CFS have also been recorded post-immunization (163) and many patients are fearful that vaccinations will worsen their already dysfunctional immune system and cause symptom exacerbation (164). More recently, ME/CFS development after the Sputnik V COVID-19 vaccination has been recorded (165). Some research about vaccination safety relates to adjuvant compounds used in some vaccinations to promote lasting immunization (166, 167). For example, aluminum hydroxide salts, often used as vaccine adjuvants, have been found to abnormally persist within immune cells at the site of previous immunization, resulting in macrophagic myofasciitis lesions (168). These inflammatory macrophage formations can result in associated microscopic muscle necrosis. While transient aberrant changes associated with aluminum hydroxide salts are acknowledged, the direct link between microscopic muscle necrosis and the mechanisms underlying ME/CFS requires further investigation. However, no indication of an increased risk of developing ME/CFS was found post-HPV vaccination (169). Additionally, ME/CFS patients were found to have similar humoral and cellular immune responses as healthy controls post-influenza vaccination (170) without worsening ME/CFS symptoms or causing adverse effects (164, 171).

Furthermore, various drug exposure has been found to trigger symptoms that are typically present in ME/CFS (172). For example, widely prescribed fluoroquinolone antibiotics are usually prescribed to treat various infections such as pneumonia and sinusitis (173–175). However, these fluoroquinolones have been found to increase tendinopathy involving oxidative stress and mitochondrial toxicity (176–180). Hence, the use of such drugs may have a multisystem effect and lead to the development of chronic illnesses such as ME/CFS.

## The complex pathophysiology of ME/CFS

As a consequence of infection and other stressors, such as poly-trauma for example (181), a state of acute inflammation and aberrant immune activation may occur. A compensatory anti-inflammatory mechanism then typically follows (59), causing an imbalance in immune responses (58, 59) combined with hypothesised autoimmunity (30, 52, 85, 182). This may lead to subsequent physiological abnormalities including gut dysbiosis and systemic inflammation, eventually resulting in a pathological clotting system, chronic endothelialitis, vasoconstriction, and hypoxia, as found in similar conditions such as heat stroke (183). Additionally, dysfunctional energy metabolism (52, 184–186) along with oxidative stress (187, 188) are also hypothesised in the development of ME/CFS. It is hypothesised that these mechanisms occur in a spiralling, progressive way, toppling the host’s homeostatic equilibrium (59).

## Gut dysbiosis

ME/CFS patients often have gastrointestinal (GI) symptoms (189) with gut inflammation (190), gut microbiome dysbiosis (57, 191) and changes in the gut microbiome (191, 192). A reduction in microbiome diversity has been identified (191), but it is likely that the microbiome composition will differ between ME/CFS patients as each patient has a unique infectious history (193). In ME/CFS, anti-inflammatory bacterial species such as *Faecalibacterium* (191, 194) and *Bifidobacterium* (191) are decreased, resulting in a decreased production of anti-inflammatory butyrate (191, 194). Butyrate is essential to maintain the mucosal barrier and immunomodulation (195), whilst having anti-inflammatory effects through downregulating pro-inflammatory cytokines (196). However, this decrease in *Faecalibacterium* is found in various disorders, and is not specific for ME/CFS (197). Similarly, some studies found an elevation in short chain fatty acids (SCFAs) butyrate, isovalerate, and valerate (198), contradicting these other articles. Conversely, proinflammatory Proteobacteria species have an elevated concentration in ME/CFS (191, 194). Proteobacteria are known as a “microbial signature of disease” (199) and one form, Enterobacteriaceae, is specifically increased in ME/CFS (191). This increased concentration of Enterobacteriaceae may result in increased transit time and IBS-like symptoms (200). It is also not yet known whether such dysbiosis leads to ME/CFS, or whether it is a consequence of the metabolic and immunologic changes that occur in the disease (201).

It is hypothesised that once an acute infection has dysregulated the host’s immune system, pathogens are capable of intracellular persistence where they escape immune surveillance (202). When metabolites and proteins expressed by these pathogens are created, they interfere with host transcription, translation, and DNA repair processes, leaving infected immune cells unable to express human metabolites. Since these pathogen proteins and metabolites are often similarly structured to ones created by the human host, such molecular mimicry makes it difficult for the host to recognise the foreign pathogen (193), resulting in the host’s immune system becoming increasingly sluggish and more susceptible to acquiring other infectious agents. This causes patients to become increasingly dysbiotic as their immune system weakens over time, and successive infections may explain the variability of symptoms experienced in ME/CFS (193).

Microbe-associated molecular patterns (MAMPs) are molecules found on bacterial surfaces (203) which interact with the immune cell receptors of the host to promote inflammation (204). Elevated levels of IgM and IgA antibodies to one potent MAMP- exotoxin lipopolysaccharide (LPS)- have been observed in ME/CFS (205), as well as higher blood levels of bacterial LPS, LPS-binding proteins, and soluble CD14 (191). LPS is a structural component in the outer membrane of many Gram-negative bacteria, and it has various immunostimulatory and procoagulant effects (206). LPS molecules have been identified as potent inflammagens (207–209) having cytotoxic and neurotoxic effects (210–214) and heightening the production of pro-inflammatory cytokines (215–218).

These inflammatory markers also indicate translocation of gut bacteria and toxins from the GI tract into the blood (191), which may result in systemic inflammation in ME/CFS (183). Specific gut inflammation (190) and gut hyper-permeability (219) have been identified, and microbe and virus communities may also persist in ME/CFS blood and brain tissue (193). Not only does the abundance of bacterial taxa correlate with the symptoms of pain and fatigue (191, 194); metabolomic results illustrate an expression of bacterial genes, rather than human (194). In essence, there appears to be a link between the microbiome, gut inflammation, and the symptoms of ME/CFS (201).

Additionally, the diverse intestinal virome that is established from birth (57) includes many prokaryotic viruses called bacteriophages (220). Bacteriophage richness was found elevated in one ME/CFS study, but it is limited owing to its small sample sise (191). Since bacteriophage predator-prey dynamics regulate the diversity and equilibrium of the bacterial microbiome (221), alterations in the virome can also cause intestinal microbial dysbiosis in different diseases (222–224) and alter microbiome homeostasis. Future studies need to include standardised techniques and analyses when doing further research into the virome as it is particularly understudied in ME/CFS (57).

**Figure 5:**
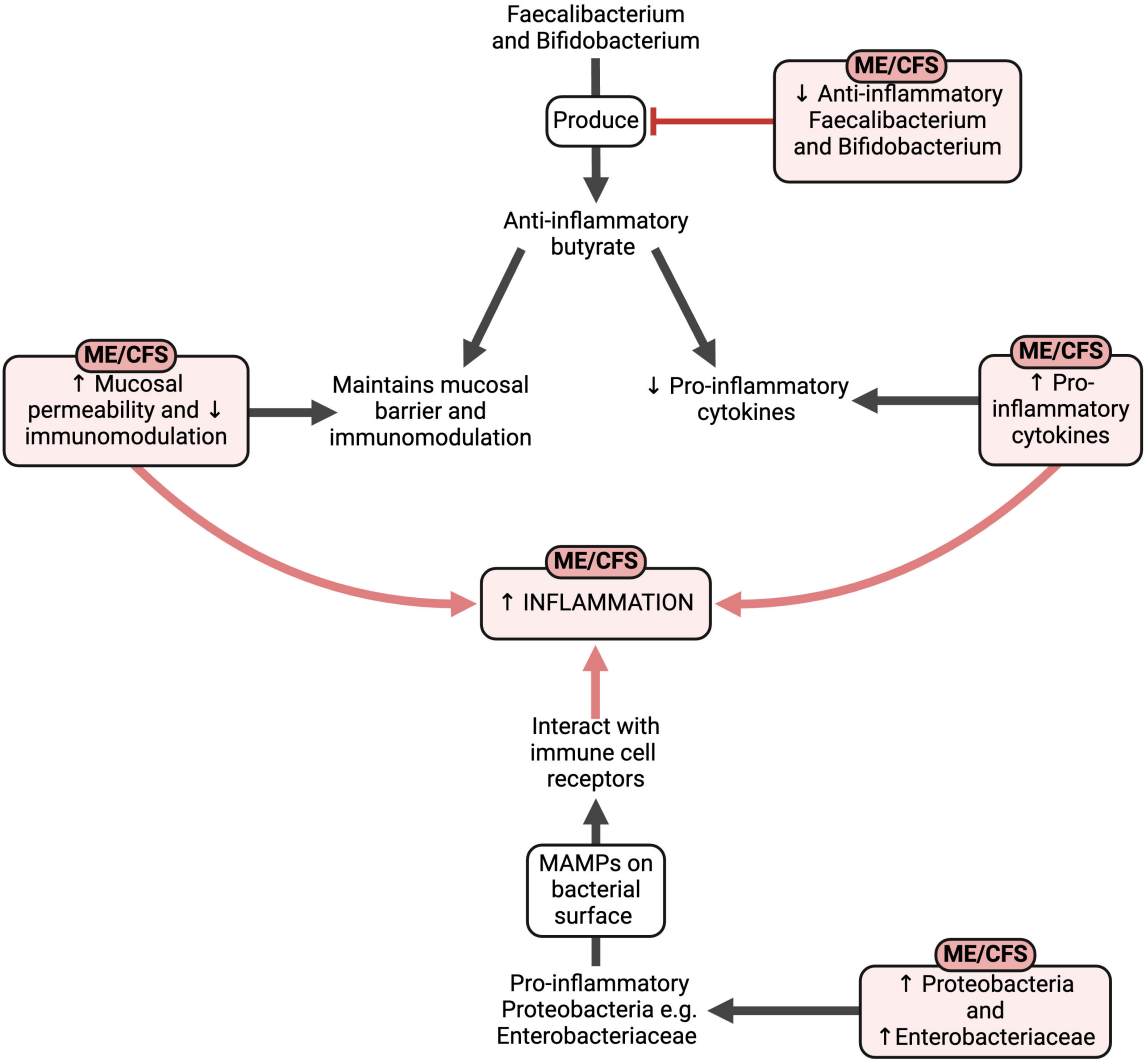
The effects of the altered gut composition in ME/CFS (57, 183, 190–196, 201, 203–209, 215–218). Created with Biorender.com. MAMP, microbe-associated molecular pattern; ME/CFS, Myalgic Encephalomyelitis/Chronic Fatigue Syndrome.

External stressors like stressful or traumatic incidents, acute infection, and toxic stressors may result in widespread and chronic systemic and even neuroinflammation, driven by a variety of inflammatory molecules in circulation. Such molecules might have their direct origins from previous infections and the resulting gut dysbiosis, as summarised in **Figure 5**.

## Systemic inflammation

Systemic inflammation (225) and neuroinflammation (45–47) are thought to play a major role in ME/CFS. Chronic inflammation is a crucial hallmark of persistent infection (193), as infections can alter proinflammatory and anti-inflammatory cytokine and chemokine expression, adjusting the inflammatory and immune responses (30). Although inflammation is reflected in selective biomarkers, traditional inflammatory markers such as C-reactive protein (CRP) and erythrocyte sedimentation rate have also shown an increased trend in ME/CFS, particularly in those with mild/moderate disease generally not raised in ME/CFS (226).

### Circulating inflammatory molecules

Early ME/CFS has been associated with elevated proinflammatory cytokines (227–230) and a distinct cytokine inflammatory profile (225, 231). Heightened circulating inflammatory cytokines are crucial in driving the development of autoimmune diseases (232). These heightened cytokines are mainly related to Th1 and Th2-driven responses, but not all studies have found consistent results. However, as ME/CFS persists over several years, it is hypothesised that the inflammatory profile (231) and plasma immune signatures change with increasing disease duration (225). Perhaps this indicates that in the early stages of ME/CFS, the immune system actively attempts to target the infectious burden (193); however as the disease progresses, pathogens in the microbiome disable the immune response and “immune exhaustion” occurs (233, 234). In essence, acute pathogens can cause chronic symptoms in ME/CFS by existing in latent forms (193).

**Figure 6:**
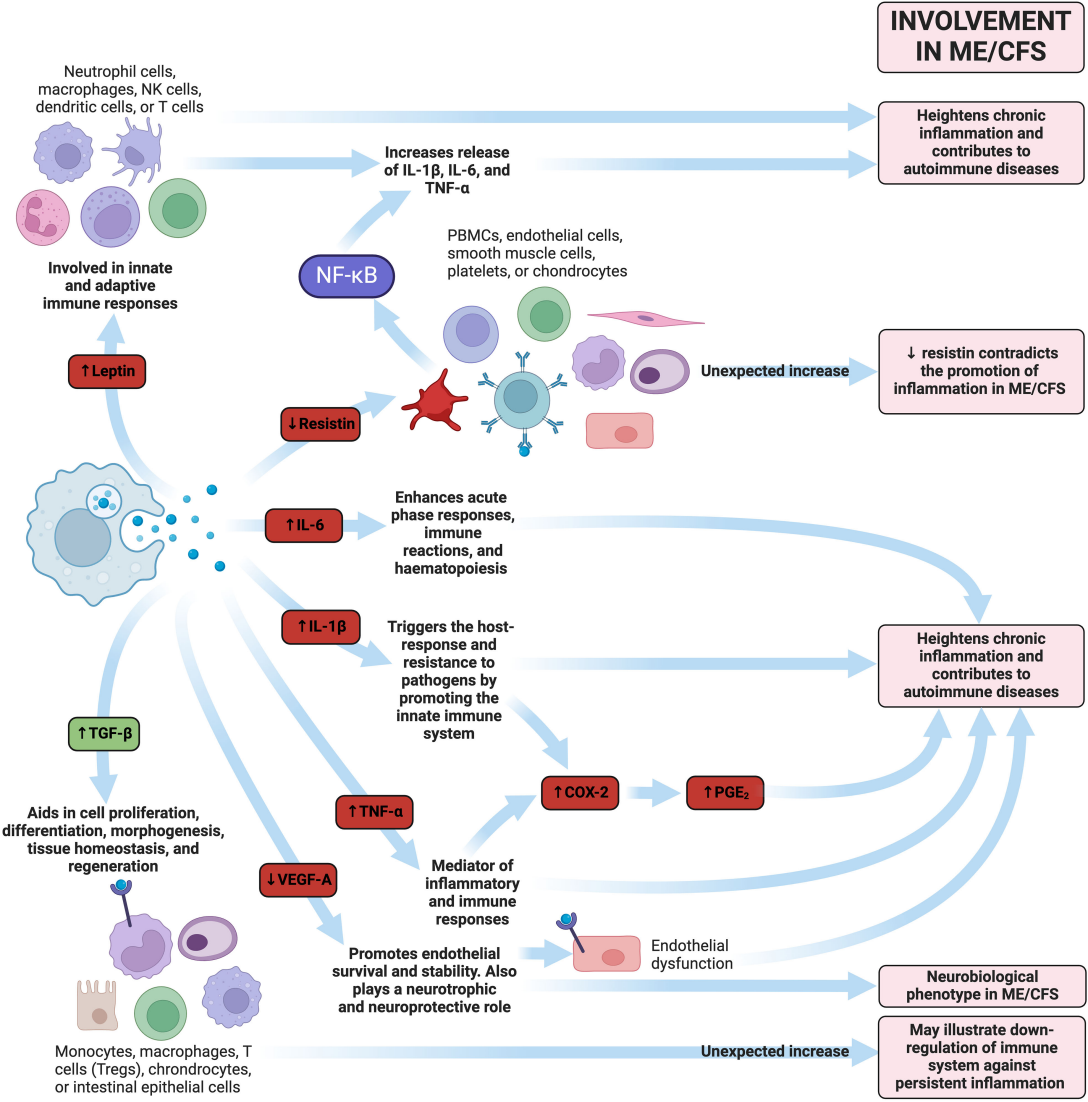
Examples of how dysregulated inflammatory molecules could play a pathological role in ME/CFS (113, 137, 225, 231, 235–247). Molecules highlighted in red represent pro-inflammatory molecules, whereas those written in green represent anti-inflammatory molecules. Created with Biorender.com. COX-2, cyclooxygenase-2; IL, interleukin; ME/CFS, Myalgic Encephalomyelitis/Chronic Fatigue Syndrome; NK, natural killer; PBMC, peripheral blood mononuclear cell; PGE2, prostaglandin-endoperoxide synthase 2; TGF-β, transforming growth factor-β; TNF-α, tumour necrosis factor-α; Treg, regulatory T cell; VEGF, vascular endothelial growth factor.

Cytokine activation has been noted in ME/CFS patients (225) and appears to increase along with disease severity (231). This suggests that patients with ME/CFS may struggle with an increasing infectious burden over time. One study noted 17 cytokines had a significant upward linear trend with the severity of ME/CFS: CCL11 (Eotaxin-1), CXCL1 (GROα), CXCL10 (IP-10), IFN (interferon)-y, interleukin (IL)-4, IL-5, IL-7, IL-12p70, IL-13, IL-17F, leptin, G-CSF, GM-CSF, LIF, NGF, SCF, and TGF-α (231). Out of these 17 cytokines, 13 are considered proinflammatory cytokines. Although these 17 cytokines are linearly correlated with ME/CFS increasing severity, they did not differ significantly between control and ME/CFS groups. This correlation of cytokine levels with severity may indicate that severity is a useful way to subgroup ME/CFS, as well as a dose-response defect in the metabolism or excretion of cytokines (231). A summary of the interactions between circulating inflammatory molecules can be found in **Figure 6**.

## Redox imbalance and oxidative stress

Inflammation is bidirectionally linked to redox imbalances (248) as inflammation produces ROS and reactive nitrogen species (RNS), whilst redox imbalances cause cellular damage, evoking an inflammatory response (249, 250). Redox imbalances (251, 252), oxidative stress (182, 187, 253–255), nitrosative stress (182), and chronic ischaemia-reperfusion injury (256) have all been found to be central to ME/CFS. Markers of oxidative stress have been found in ME/CFS and, importantly, correlate with symptom severity (251) (**Table 1**).

**Table 1:** Markers of oxidative stress present in ME/CFS (adapted from Paul et al., 2021) (188, 257–296).

Biomarkers illustrating elevated oxidative stress	Levels in ME/CFS	Effect in ME/CFS
Peroxide	Elevated and increases with symptom severity (257).	Hydrogen peroxide is produced by vascular and inflammatory cells, inducing oxidative stress (258).
Superoxide and superoxide dismutase (SOD)	Elevated and increases with symptom severity (257) and decreased SOD activity and expression (259).	The mitochondrial electron transport chain produces superoxide under physiological conditions, elevating oxidative stress (260). SOD protects cells against oxidative stress in mitochondria (261). Hence, this beneficial mechanism is reduced in ME/CFS.
Malondialdehyde (MDA)	Increased (188, 262–264).	Increased free radicals causing an excess production of MDA (265).
Thiobarbituric acid reactive substances (TBARs)	Increased (263, 264, 266) and correlate with symptom severity (267).	TBARs are generated through lipid peroxidation (268).
Iron and heme metabolism	Elevated heme and lowered serum transferrin (263, 269).	Heme catalyses the formation of ROS (270). Decreased transferrin indicates excess iron (271).
Homocysteine	Increased cerebrospinal fluid levels (272).	Elevated homocysteine promotes oxidative stress in vascular cells through ROS formation (273).
Nitric oxide (NO)	Elevated NO (259, 274) and heightened citrulline (a product of arginine metabolism by nitric oxide synthase (NOS)) (275).	During inflammation, the over production of NO by the vasculature contributes to oxidative stress (276).
Oxidised LDL	Increased (188, 263).	Oxidation of LDL particles and excessive ROS generation are present in oxidative stress (277).
Neutrophil response	In ME/CFS, an initial aggressive neutrophil response occurs, followed by neutropenia and a lowered oxidative, ending off with neutrophil exhaustion burst (278).	Neutrophils generate ROS and RNS in an ‘oxidative burst’ to induce neutrophil extracellular traps (NETs) (279–282).
Glutathione (GSH)	Lowered GSH levels in the cortex of the brain and plasma (188, 283), catalase (259), glutathione peroxidase (259), and glutathione reductase activities in erythrocytes (259).	GSH prevents cell damage during oxidative stress (284, 285), meaning this beneficial mechanism is reduced in ME/CFS.
Vitamin C/ ascorbate	Low plasma levels (267, 286).	Vitamin C usually behaves as a ROS scavenger (287), but this mechanism is lowered in ME/CFS.
Vitamin E	Reduced serum levels of vitamin E (188, 263, 264) correlating with severity of symptoms and the levels increase with remissions (264, 288). Vitamin E is also lowered in paediatric cases (286).	Vitamin E usually behaves as a ROS scavenger (289), but this mechanism is lowered in ME/CFS.
Alpha-tocopherol	Decreased levels (288, 290).	Alpha-tocopherol acts as a free radical scavenger, but these mechanism is lowered in ME/CFS (291).
Cysteine	Low levels of cysteine and oxidized cysteine in ME/CFS, but elevated levels of cysteine and methionine (methionine sulfoxide) in the PBMCs of ME/CFS individuals (292).	Cysteine directly scavenges free radicals (293), meaning this beneficial mechanism is reduced in ME/CFS.
NAD metabolism	Nicotinamide phosphoribosyl transferase levels increased (294).	NAD is phosphorylated by NAD kinase to form NADP (295). From there, it is reduced to NADPH by NADP dehydrogenase. NADPH acts as an antioxidant to neutralise high levels of ROS (296). Interestingly, this beneficial mechanism is increased in ME/CFS.

LDL, low-density lipoprotein; ME/CFS, Myalgic Encephalomyelitis/Chronic Fatigue Syndrome; PBMC, peripheral blood mononuclear cell; ROS, reactive oxygen species.

Nitrosative stress is also present, as illustrated by markers of nitrosative stress such as increased NOS and NO (182, 297), peroxynitrite (182, 297), elevated NF-κβ production (182), and nitrate after exercise (182, 297). Even though NO is physiologically critical to vasodilation and neurotransmission (251), excess NO and RNS are damaging as they directly attack antioxidant enzymes such as catalase (298), promoting redox imbalance.

When muscle afferents are triggered by muscle fatigue, this triggers the production of heat shock proteins (HSPs) (299). HSPs protect muscle cells against any deleterious effects of ROS generated during exercise by activation of antioxidants (300, 301). In turn, the elevated antioxidant levels elevate the levels of plasma HSPs. However, prolonged activation of muscle afferents by oxidative stress due to low-grade exercise results in reduced HSP production (266). The formation of HSPs in ME/CFS individuals is reduced (302), and the responses of plasma HSP27 and HSP70 are delayed or lowered, while resting levels of plasma HSP70 are also decreased (303). Hence, this impaired HSP production (302) combined with oxidative and nitrosative stress, and low-grade inflammation could explain the exercise intolerance and muscle dysfunction seen in ME/CFS patients (303, 304).

## Dysfunction of the vasculature, endothelium, and coagulation

Since persistent inflammation and immune cell activation is present in ME/CFS (305), vascular changes and endothelial damage (306, 307) will coexist due to the interplay between inflammation and vascular alterations. The altered autoregulation of blood flow cannot meet the metabolic demand of tissues in ME/CFS, leading to tissue hypoxia and subsequent ischaemia/reperfusion injury with its associated symptoms and signs (29).

### Endothelial damage

Endothelial cells are important regulators of the immune system (308); endothelial dysfunction can promote oxidative stress and inflammation (309). The abnormal immune responses present in ME/CFS are thought to impact endothelial cell function (29) and patients show signs of endothelial dysfunction (306, 310). Endothelial dysfunction has been demonstrated *in vivo* (306, 311), in the large vessels of ME/CFS patients (307, 311) through flow-mediated dilation (FMD) and in the microcirculation through post-occlusive reactive hyperaemia.

FMD measures the dilation of blood vessels triggered by the release of NO from endothelial cells in response to shear stress (307, 311). It was also found that ME/CFS patients are unable to dilate their vessels adequately by endothelium-independent vasodilation when given sublingual nitroglycerin to promote relaxation of the vessels. Additionally, myocardial infarction associated transcript (MIAT) was found to be upregulated in PBMCs of ME/CFS patients, indicating endothelial dysfunction (312). Additionally, microclot presence (313) in ME/CFS may also cause damage to the endothelium. However, the endothelial damage observed in ME/CFS patients does not appear to correlate with the usual markers of endothelial dysfunction seen in cardiovascular disease such as increased levels of symmetric dimethylarginine (SDMA), asymmetric dimethylarginine (ADMA), and high-sensitivity C-reactive protein (hs-CRP), and reduced levels of arginine and homoarginine (311). This may suggest that a different mechanism is at play, which could relate to the abnormal immune response present in ME/CFS (311). Endothelial dysfunction can result in capillary leakage, accelerated inflammation, hypercoagulation, platelet aggregation, and decreased vascular tone (314).

**Figure 7:**
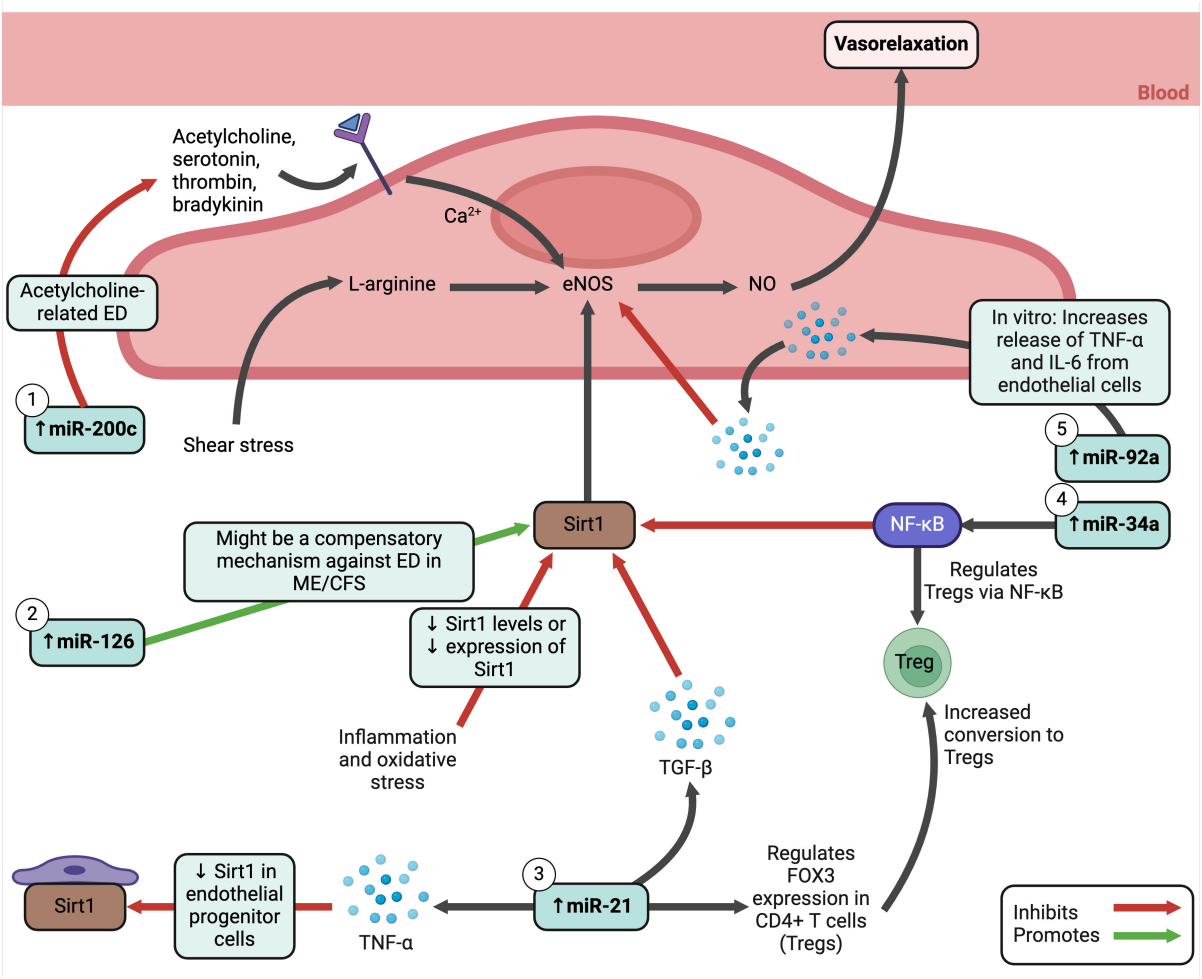
Elevated MicroRNAs in ME/CFS and how they influence endothelial cell functioning (305, 316, 319–328). Created with Biorender.com. ED, endothelial dysfunction; eNOS, endothelial nitric oxide synthase; Fox3, transcription factor forkhead box protein 3; IL, interleukin; ME/CFS, Myalgic Encephalomyelitis/Chronic Fatigue Syndrome; miRNA, micro ribonucleic acid; NF-κB, nuclear factor kappa B; NO, nitric oxide; Sirt1, silent information regulator 1; TGF-β, transforming growth factor beta; TNF-α, tumour necrosis factor alpha; Treg, regulatory T cell.

MicroRNAs (miRNAs) are important to maintain endothelial function and altered miRNA profiles are often used to predict, diagnose, and monitor diseases (315). Studies have revealed interesting miRNA changes in ME/CFS. Silent information regulator 1 (Sirt1), an anti-inflammatory and anti-oxidative protein (316), increases the production of NO by activating endothelial NOS (eNOS) in endothelial cells in response to shear stress (317). The NO released by endothelial cells controls the vascular system to ensure sufficient blood and oxygen reaches tissues throughout the body. During inflammation and oxidative stress, eNOS uncoupling or reduction can occur (318), as well as decreased activity or expression of Sirt1 (316). In ME/CFS, a set of plasma miRNAs known to modulate the Sirt1/eNOS axis were analysed, showing elevation in miR-21, miR-34a, miR-92a, miR-126, and miR-200c (305). These five miRNAs have also been found increased in PBMCs from different cohorts. The functions of these miRNAs are illustrated in **Figure 7**; they relate to endothelial function signalling pathways, including oxygen regulation and oxidative stress. Hence, miRNAs may serve as a potential biomarker in ME/CFS, although they do not correspond with disease severity (305).

### Autonomic dysfunction, vasoconstriction, and hypoxia

Many ME/CFS patients have a unique cardiovascular situation (58) and changes indicative of autonomic dysfunction (329–331). One cluster analysis of 131 ME/CFS patients diagnosed according to the Fukuda criteria revealed that 34% of patients experienced sympathetic symptoms with dysautonomia (and were associated with more severe disease severity), 5% from sympathetic symptoms alone, 21% parasympathetic, and 40% struggled with sympathovagal balance (331). However, studies assessing autonomic dysfunction in ME/CFS are equivocal (329). Since heart rate parameters often reflect changes in autonomic function, one meta-analysis analysed 64 studies to assess differences in heart rate parameters in ME/CFS (329). It was concluded that, in comparison with controls, patients with ME/CFS have a higher resting heart rate, lower maximal/peak heart rate, higher heart rate responses to head-up tilt testing and moving from sitting to standing, and a lower heart rate at submaximal exercise threshold. Moreover, the resting heart rate variability (HRV) parameters also differed in ME/CFS patients, with a higher low frequency power of HRV (LFP) and a lower high frequency power of HRV (HFP). This corresponds with other studies that suggest a decreased HRV is present in ME/CFS, indicative of a chronically high sympathetic tone (78, 330, 332–344). Hence, the results of the meta-analysis may indicate reduced vagal modulation in ME/CFS, along with increased sympathetic modulation of heart rate (329).

**Figure 8:**
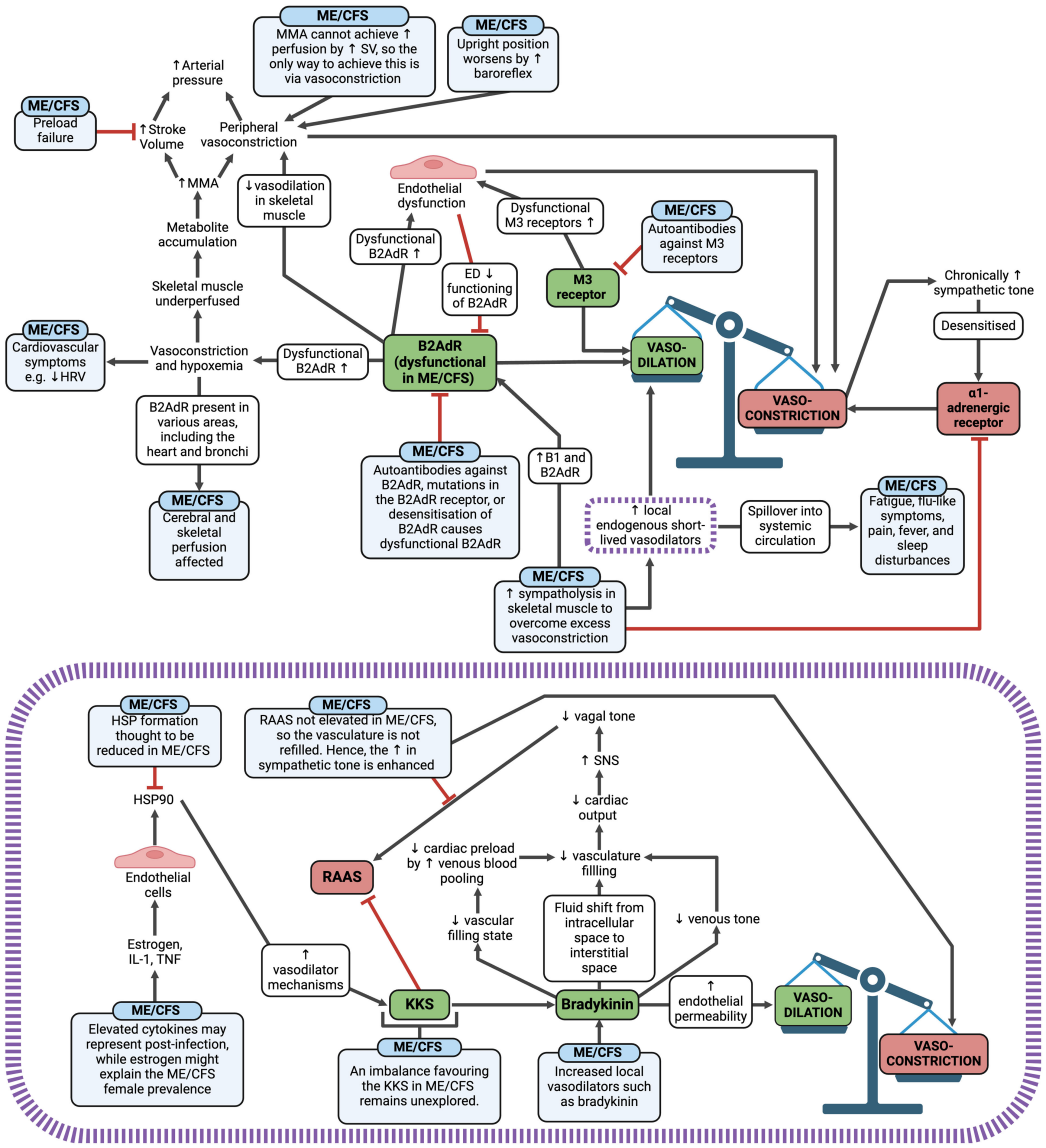
Vasoconstriction and hypoxia mechanisms in ME/CFS and an example of the potential effect of the local vasodilator bradykinin in ME/CFS (illustrated in the purple box) (58, 78, 183, 330, 332–348). Created with Biorender.com. B2AdR, beta-2 adrenergic receptor; ED, endothelial dysfunction; HRV, heart-rate variability; HSP, heat shock protein; IL, interleukin; KKS, kallikrein-kinin system; M3, muscarinic acetylcholine receptor M3; ME/CFS, Myalgic Encephalomyelitis/Chronic Fatigue Syndrome; MMA, muscle metaboreflex activation; RAAS, renin-angiotensin-aldosterone system; SNS, sympathetic nervous system; SV, stroke volume; TNF, tumour necrosis factor.

Autonomic changes in ME/CFS are likely to cause an overall effect of vasoconstriction and resultant hypoperfusion (58), which is central to the pathology of ME/CFS (183) (**Figure 8**). Additionally, vascular dysfunction, endothelial dysfunction, and microclot presence may also promote vasoconstriction and tissue hypoxia. When skeletal muscle is hypoperfused, metabolites accumulate and trigger muscle metaboreflex activation (MMA) (58) to increase the arterial pressure. If preload failure exists in ME/CFS, it is likely that MMA cannot achieve a sufficient rise in perfusion by increasing stroke volume; hence the only way to increase the blood pressure is via further vasoconstriction. This is worsened when ME/CFS patients are upright, as the baroreflex is activated (345), further promoting vasoconstriction in skeletal muscle. Additionally, when B2AdR is dysfunctional, it aggravates the situation by inhibiting vasodilation in skeletal muscle (58). Therefore, three factors cause excessive vasoconstriction: B2AdR dysfunction, excessive baroreflex activation due to hypovolemia, and excessive MMA activation due to the poor metabolic situation. With a chronically high sympathetic tone, there is then a need for sympatholysis in skeletal muscles to prevent vasoconstriction via the α1-adrenegeric receptors (58). Sympatholysis enhances the release of local endogenous, short-lived vasodilators such as adenosine, ATP, prostaglandins, prostacyclin, bradykinin, and protons to act as a compensatory mechanism (58). However, if these vasodilators enter the systemic circulation, they could contribute to ME/CFS symptoms such as fatigue, flu-like symptoms, pain, fever, and sleep disturbances, similar to the effects observed when vasodilators spill over in dysmenorrhea (346). An example of this effect can be observed with bradykinin (purple box within **Figure 8**) (347, 348), and preload failure experienced in ME/CFS may be correlated with the endogenous vasodilator substances produced in skeletal muscles.

### Abnormal coagulation: the role of microclots and inflammagens in circulation that cause pathological clotting

It has been hypothesised that hypercoagulation plays a significant role in ME/CFS (349–351), but there are still discrepancies between studies (352). Hence, there is a need to investigate this matter in greater detail. In one study, ME/CFS blood samples displayed significant hypercoagulability in thromboelastography (TEG) analyses of both whole blood and platelet poor plasma (313). Platelet hyperactivation was also observed, as well as the presence of microclots containing fibrinogen and amyloid proteins. The area of these “fibrinaloid” microclots was found to be greater in ME/CFS samples in comparison to healthy controls (313).

**Figure 9:**
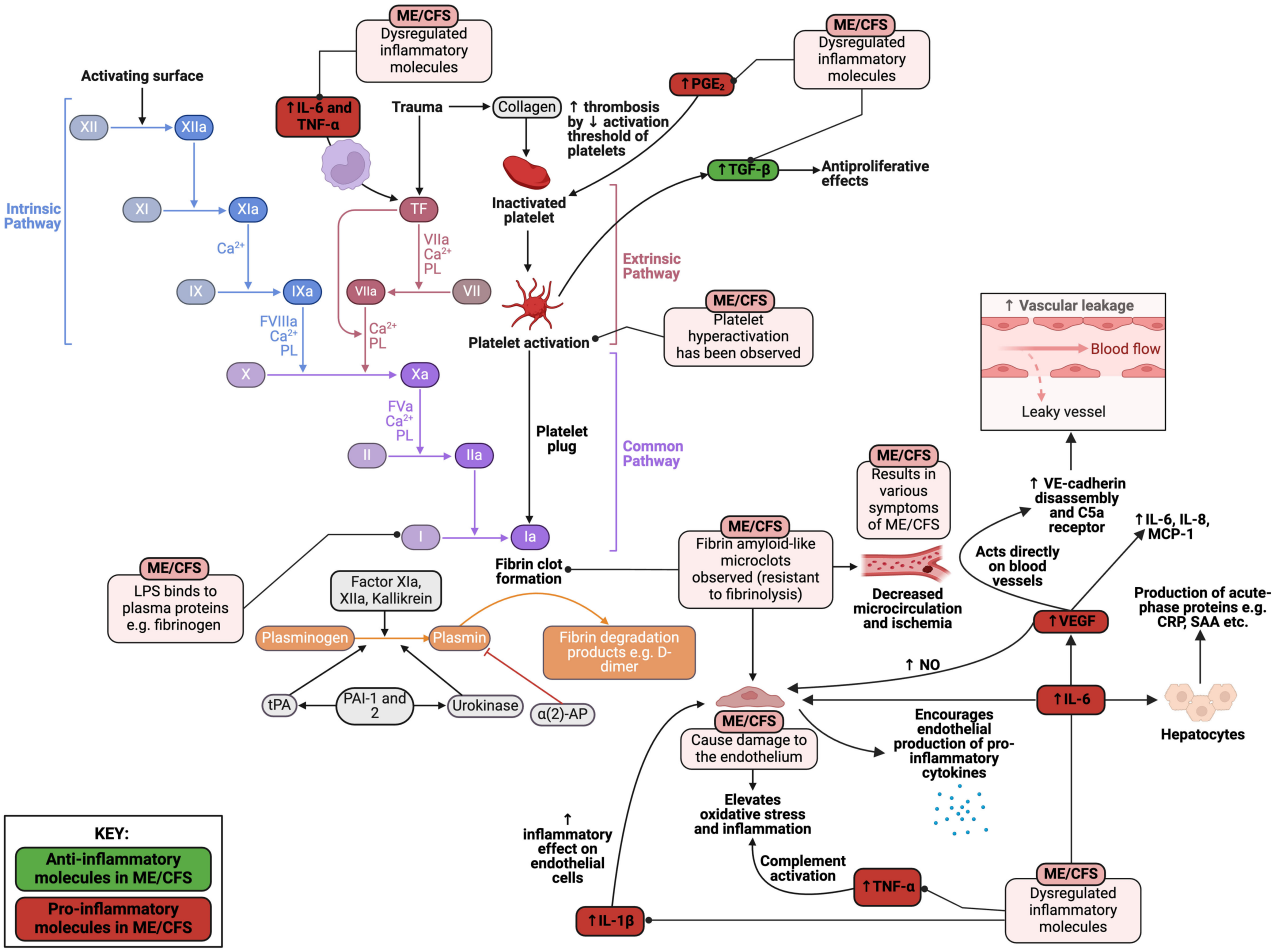
Pathological alterations in the coagulation cascade present in ME/CFS (191, 205, 313, 349–353). Created with Biorender.com. α (2)-AP, alpha-2-antiplasmin; CRP, C-reactive protein; IL, interleukin; LPS, lipopolysaccharide; MCP-1, monocyte chemoattractant protein-1; ME/CFS, Myalgic Encephalomyelitis/Chronic Fatigue Syndrome; NK, natural killer; NO, nitric oxide; PAI-1, plasminogen activator inhibitor 1; PBMC, peripheral blood mononuclear cell; PGE_2_, prostaglandin-endoperoxide synthase 2; SAA, serum amyloid A; TF, tissue factor; TGF-β, transforming growth factor beta; TNF-α, tumour necrosis factor alpha; tPA, tissue plasminogen activator; VEGF, vascular endothelial growth factor.

As mentioned previously, elevated levels of LPS molecules in ME/CFS patients have been documented (191) with increased levels of IgM and IgA antibodies to LPS in serum (205). LPS can directly and acutely bind to plasma proteins such as fibrinogen to promote the formation of these “fibrinaloid” microclot deposits (353). Hence, not only does LPS induce chronic inflammation via cytokine production; it also has a hypercoagulatory effect by binding to plasma proteins. Since these “fibrinaloid” microclots are usually more resistant to fibrinolysis, they are hypothesised to linger in the circulation and have extended contact with the endothelium (313). Hence, the microclots may result in decreased circulation and blockage of the microcapillaries, resulting in ischemia and therefore many symptoms of ME/CFS, as seen in **Figure 9**.

## Neuroinflammation

**Figure 10:**
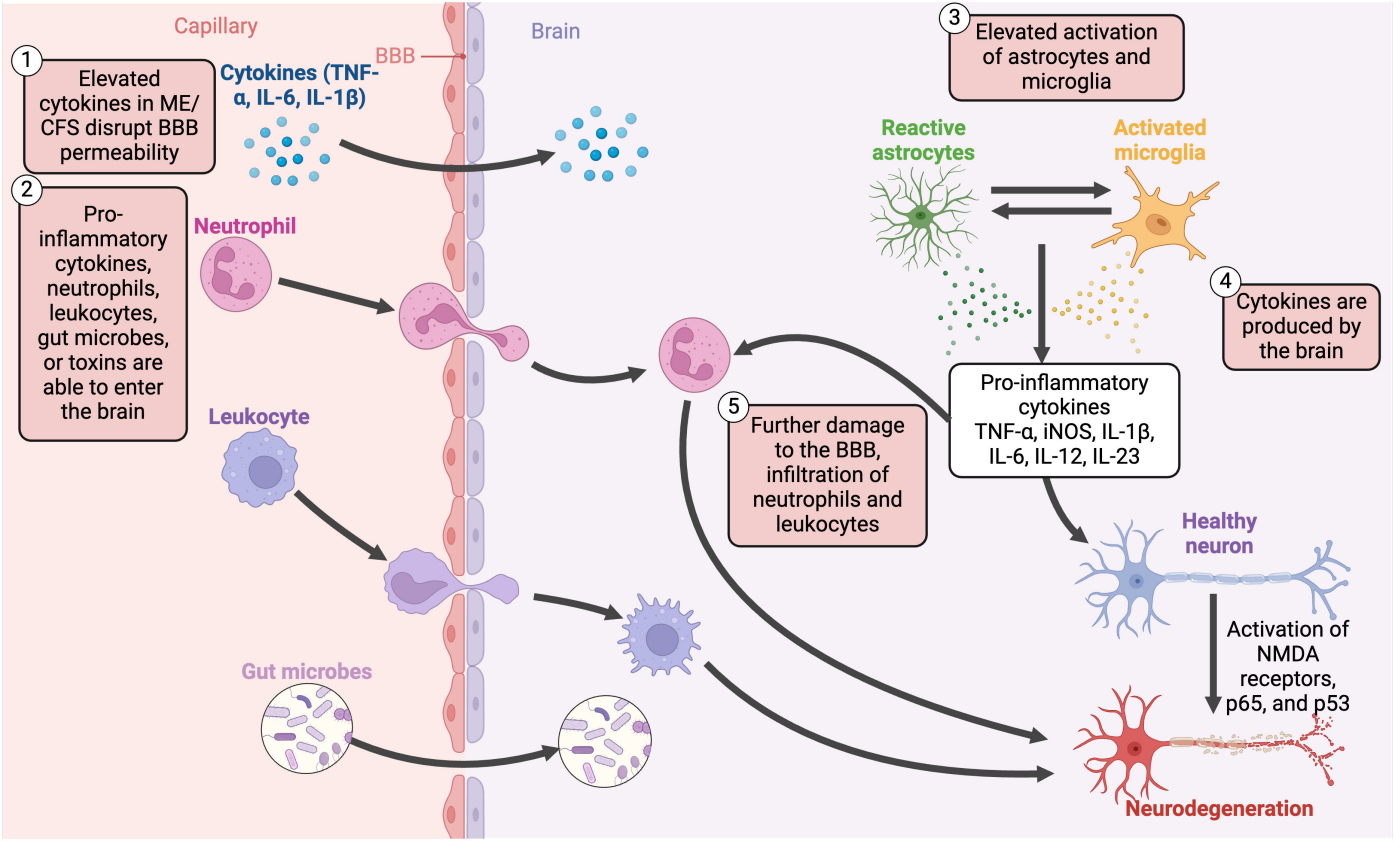
Disruption of the blood-brain barrier, the translocation of pro-inflammatory cytokines, and chronic activation of various non-neuronal cells contribute to neuroinflammatory mechanisms in ME/CFS (45, 47, 52, 201, 251, 354, 355). Created with Biorender.com. BBB, blood-brain barrier; IL, interleukin; iNOS, inducible nitric oxide synthase; ME/CFS, Myalgic Encephalomyelitis/Chronic Fatigue Syndrome; NMDA, N-methyl-D-aspartate; TNF-α, tumour necrosis factor alpha.

PET imaging has displayed elevated cytokines in the spinal cord and brain (46, 354), as well as increased activation of astrocytes (46, 354) and microglia (46, 47, 354). Although cytokines are mainly produced by the immune system, there is also an unclear link between neuroinflammation and these peripheral proinflammatory cytokines (46) and some cytokines may also be produced in the CNS (201). These cytokines can promote cognitive dysfunction as they are able to disrupt the blood-brain barrier (BBB), allowing proinflammatory cytokines (52), cells (such as dendritic, B cells, and T cells) (52), and gut microbes or toxins (355) to translocate into the brain and promote inflammation (**Figure 10**). One *in vitro* study found that EBV dUTPase altered the expression of 34 genes with roles related to BBB integrity (137). Hence, EBV UTPase may alter the synaptic structure and function in ME/CFS, as well as dysregulate neuronal communication, influencing cognitive processes.

Such inflammatory changes potentially cause impaired autoregulation of blood flow (29), hypoperfusion in the brain stem (356–358), and brain tissue hypoxia (22, 23, 26, 29–101, 103, 109, 113, 127–234, 248–256, 266, 297–318, 329–359).

Tilt tests of ME/CFS patients have shown a reduced cerebral blood flow (360) and elevated intracranial pressure (361, 362). These changes may cause disordered sympathetic and parasympathetic activity (158, 360) as well as PEM and cognitive dysfunction (29). Likewise, such neuroinflammation can interact with neurotransmitters, elevate procoagulant activity and thrombosis, and cause endothelial damage, resulting in neurovascular coupling (NVC) dysfunction (363).

## Impaired energy metabolism in ME/CFS

Even though an exact metabolic phenotype has not been established (185), a stressed metabolism is present in ME/CFS. When threats to homeostasis occur such as infection, hypoxia, and starvation (364, 365), they involve immune and inflammatory processes that influence energetics and metabolism (366, 367). In the case of ME/CFS, this energy strain may be a consequence of exertion-sensitive tissue hypoxia, leading to systemic patterns of metabolic adaptation and compensation (185). It is thought that over time, the disease shifts from an early hypermetabolic state to a hypometabolic state with decreased metabolites (251) and reduced energy production (59), correlating with patient clinical profiles (185).

Mitochondrial dysfunction is a prominent feature in ME/CFS (60, 368–375). It is known to exacerbate inflammation and redox imbalances (251) by triggering the NLR family pyrin domain containing 3 (NLRP3) inflammasome (251), increasing the release of inflammatory cytokines such as IL-1β and IL-18 (376). Additionally, damaged mitochondria release mtDNA into the cytosol, activating the innate immune system via damage-associated molecular patterns (DAMPs) (251, 377), modulating both innate and adaptive immune responses (377–379). Moreover, mitochondrial damage elevates ROS levels (380), which can in turn damage mtDNA and proteins that may be involved in the electron transport chain (ETC), resulting in lowered ATP production and decreased energy levels (381).

This mitochondrial dysfunction is a result of structural and functional changes. Structural mitochondrial abnormalities have been seen in muscle biopsies from ME/CFS patients (382), but this is not observed in other studies (383). More condensed mitochondrial cristae were observed in blood cells from ME/CFS patients, but the mitochondrial crista length, sise, shape, density, membrane potential, and enzymatic activities of the complexes inside the ETC remained intact (384). In CD4+ T cells, mitochondrial mass was also not altered in ME/CFS (254).

Metabolic abnormalities are evident in ME/CFS, such as reduced mitochondrial respiratory function in ME/CFS neutrophils (371, 375) and PBMCs (385). Although mitochondrial respiration was noted as unchanged in resting and stimulated CD4+ and CD8+ T cells, CD8+ T cells were found to have a reduction in proton leak, ATP synthesis, and mitochondrial membrane potential (254). Decreased ATP production has also been noted in lymphoblasts (386) and PBMCS (387). Additionally, decreased glycolysis has been observed in CD8+ T cells at rest and after activation (254), CD4+ T cells at rest (254), blood and urine samples (187), and PBMCs (388). Similarly, a decreased glycolytic reserve has been found in NK cells from ME/CFS patients (389) and metabolomic analyses have revealed compromised ATP production via the tricarboxylic acid (TCA) cycle (389). These dysfunctions may arise from impaired pyruvate dehydrogenase (PDH) function identified in muscle cells (390) and serum (184), as well as reduced plasma coenzyme Q10 (CoQ10) levels in ME/CFS blood and plasma (372, 391) that is inversely associated with fatigue severity (391). Abnormal oxidative phosphorylation may also occur in neutrophils (371, 375) and PBMCs (388).

If such aerobic metabolism is impaired, the body switches to anaerobic production which generates nominally 18 times less ATP per glucose molecule and produces more lactic acid (392). Two-day cardiopulmonary exercise tests (CPET) have practically demonstrated this reduced anaerobic threshold in ME/CFS (393, 394). Some clinical studies have found elevated lactate levels (395, 396) in ME/CFS patient blood, cerebrospinal fluid, and muscles, suggesting there is reduced oxidative phosphorylation and a switch to anaerobic glycolysis (392, 397–399). Moreover, elevated serum lactate dehydrogenase (LDH) has been observed (400), suggestive of tissue destruction, along with elevated lactic acid levels (47, 397, 401). However, the relationship between lactic acid levels and ME/CFS is still being investigated, as another study reported reduced lactic acid levels in urine, along with lower pyruvate and alanine (187). Additionally, it is worth considering that heightened lactic production, in conjunction with its clearance during rest and exercise, may function in a compensatory manner with lactic acid acting as an energy source. Skeletal muscle acidosis and dysregulated protons have also been found in patients during or after exercise (401–404).

On the other hand, there is also altered utilisation of amino acids and fatty acids as catabolic fuel (184, 186). These studies hypothesised that this impaired PDH activity in ME/CFS may result in increased amino acid utilisation as an energy source. Metabolomic studies have displayed this altered cellular energetics, but the affected metabolites differ between studies (186, 269, 405–407). Some affected metabolites link to amino acid metabolism and changes in key pathways such as amino acid metabolism has been observed (184, 187, 408, 409). Changes in blood glucose and lipids indicate a metabolic shift (405, 406, 410, 411) as there are reduced levels of acyl-carnitine and fatty acids (412), and amino acids from the urea cycle (187, 408). In essence, energy fuel storage, mobilisation, and utilisation may be altered in ME/CFS patients.

This energy impairment likely results in a hypometabolic state as the illness progresses (59, 401, 407, 413), called the “cell danger response” (414–416). This state may be secondary to a persisting stressor, such as a redox imbalance (251), persistent infection (201, 251), injury (201), insufficient nutrients (201), cold temperatures (201), or it could be due to a defect in the “switch” that turns off this protective state (251). However, the reduced blood flow and resulting ischaemia may also act as a stressor itself. This cell danger response is an evolutionary adaptation enabled to protect the cells and host from harm (414–416). At the level of the organism, this is called the “integrated stress response” (ISR) (417). In this process, non-essential energy-consuming mechanisms are reduced, allowing energy molecules to be used for mechanisms that are crucial for viability. Neuroinflammation or fever is presumed to also trigger ISR, as autoantibodies may target neural or immune systems and cause inflammation elsewhere (418). Since redox imbalance is a mechanism involved in ME/CFS, it may be an indication of systemic inflammation in response to persistent infection or injury (251).

Even though mitochondrial dysfunction appears evident in ME/CFS, the causes of such dysfunction are speculative (251) and there is inconsistent evidence correlating mitochondrial dysfunction and ME/CFS (254, 283, 386, 389, 419). In addition, mitochondria modulate intracellular calcium homeostasis and immune regulatory pathways (420, 421), which means these pathways may too be compromised in ME/CFS.

## Hormonal alterations

Since stress has been described as a potential trigger for ME/CFS (422) and cause of symptom flare-ups (423, 424), it is likely that the HPA axis- the neurobiological stress system- may be implicated in ME/CFS (53, 425, 426), as well as abnormalities in growth hormone (GH) secretion and dysfunctional adrenergic metabolism (255). Immune and inflammatory responses in the blood are mediated by the HPA axis to prevent any autoimmune alterations (63). If the presence of an initial stressor is prolonged, the HPA axis will become chronically activated and trigger the overproduction of cortisol which, over time, will result in a reduction in cortisol levels (427). It is thought that hormonal changes such as this hypocortisolism may result in symptoms of fatigue experienced by patients (255, 428, 429). A potential negative feedback loop, often colloquially referred to as a “stress crash” or “adrenal burnout”, is likely to develop. However, it is important to note that “adrenal burnout” is not an official medical diagnosis, and the mechanisms involved may not directly involve the adrenals experiencing complete exhaustion (63). Chronic HPA activation elevates cortisol production, lowering immune responses and the production of proinflammatory cytokines. However, the HPA axis will then respond to these heightened cortisol levels by decreasing the production of cortisol over time. This will reduce the protection provided by the HPA axis, attenuating immune and inflammatory changes, and leaving ME/CFS patients more vulnerable to minor stressors (429). It is also possible that HPA sensitivity rather than HPA axis dysfunction exists in patients (63).

In one ME/CFS study, significantly lower levels of adrenocorticotropin (ACTH)/cortisol were found (430). GH peak/insulin-like growth factor-1 (IGF-1) were also significantly reduced in severe ME/CFS patients compared to controls and ME/CFS patients with mild disease. GH/IGF-1, and particularly IGF-1, play various roles in neurons such as neuroprotection, mitochondrial protection, antioxidant defence, and reduction in CNS inflammation (431). Hence, a reduction in these hormones would result in these beneficial mechanisms being reduced (430). Similarly, impaired GH release after exposure to dexamethasone was also found in patients exposed to organophosphates (153). GH is secreted from somatotrophs in the anterior pituitary gland and is influenced positively by growth hormone-releasing hormone (GHRH) and inhibited by somatostatin. Exercise and stress with B-adrenergic stimuli decrease GH secretion by elevating somatostatin tone. Hence, impaired release of GH in these toxin-exposed patients may suggest lowered responsivity of CNS type II glucocorticoid receptors (153).

Serotonin also plays a role in the CNS and controls many stress mechanisms such as the HPA axis through stimulation of corticotropin-releasing hormone (CRH) (432). It is proposed that the production and recycling of dopamine and serotonin is implicated in ME/CFS, which could also be triggered by the EBV dUTPase protein (137). Although an imbalance of these hormones is likely present in ME/CFS, it is unclear whether they are found in elevated or lowered concentrations. One article proposed that excessive serotonin levels could explain classic symptoms of ME/CFS (433), as it would promote the release of excess CRH, and therefore cause chronic reactivation of the HPA axis (434). Furthermore, this excess serotonin could eventually lead to dysregulation of its production (435). Excess serotonin can result in decreased control of various functions, including dysfunctional muscle contraction, migraines, sleep issues, dyspnea, hyperalgesia, and cognitive dysfunction (435). Heightened serotonin levels can also promote the release of dopamine and norepinephrine, resulting in changes to memory, GI problems, mood, and blood coagulation (436). However, lowered dopamine levels are associated with fatigue (437), a commonly present symptom of ME/CFS.

Exposure to toxins such as organophosphates, which are cholinesterase inhibitors, prolongs and amplifies the effects of acetylcholine (153). Acetylcholine is responsible for mood regulation, psychomotor activity, and sleep (438), by activation of central muscarinic receptors, rather than nicotinic receptors (153). Acetylcholine is also known to promote GH secretion (439). However, patients exposed to organophosphates experience a heightened GH response to pyridostigmine (153). It is possible that the somatotrophs developed increased sensitivity to GHRH as pyridostigmine causes intermediate stimulation of GHRH. Alternatively, the more supported hypothesis is that there could be hyper-responsivity of the cholinergic receptors at a hypothalamic level, causing a greater decrease in somatostatin tone, and elevated GH release from the anterior pituitary gland (153).

Various symptoms seen in ME/CFS, such as changes in body weight, appetite, fluid retention, and irregular menstruation, are also observed in hypothalamic dysfunction (440). Dysfunctional hypothalamic function can be seen in ME/CFS patients in the form of up-regulation of hypothalamic 5-hydroxytryptamine (5-HT) receptors (441) and abnormal arginine/vasopressin responses to deprivation tests and water loading (440). However, when the 5-HT-releasing agent D-fenfluramine is used in patients experiencing a neurobehavioral syndrome after exposure to organophosphates, elevated sensitivity of central 5-HT receptors is observed (442).

It is also possible that hypothalamic/pituitary autoimmunity may be present in ME/CFS- particularly in the more severe cases- as antipituitary and antihypothalamic antibodies have been identified (430). Additionally, if gut permeability is indeed increased in ME/CFS patients, microbes and antigens may be able to cross the epithelial barrier into surrounding tissue and blood, potentially crossing the BBB and altering the HPA axis (443). Hence, GI dysbiosis may also promote HPA axis activation (63).

## Immune dysfunction

**Figure 11:**
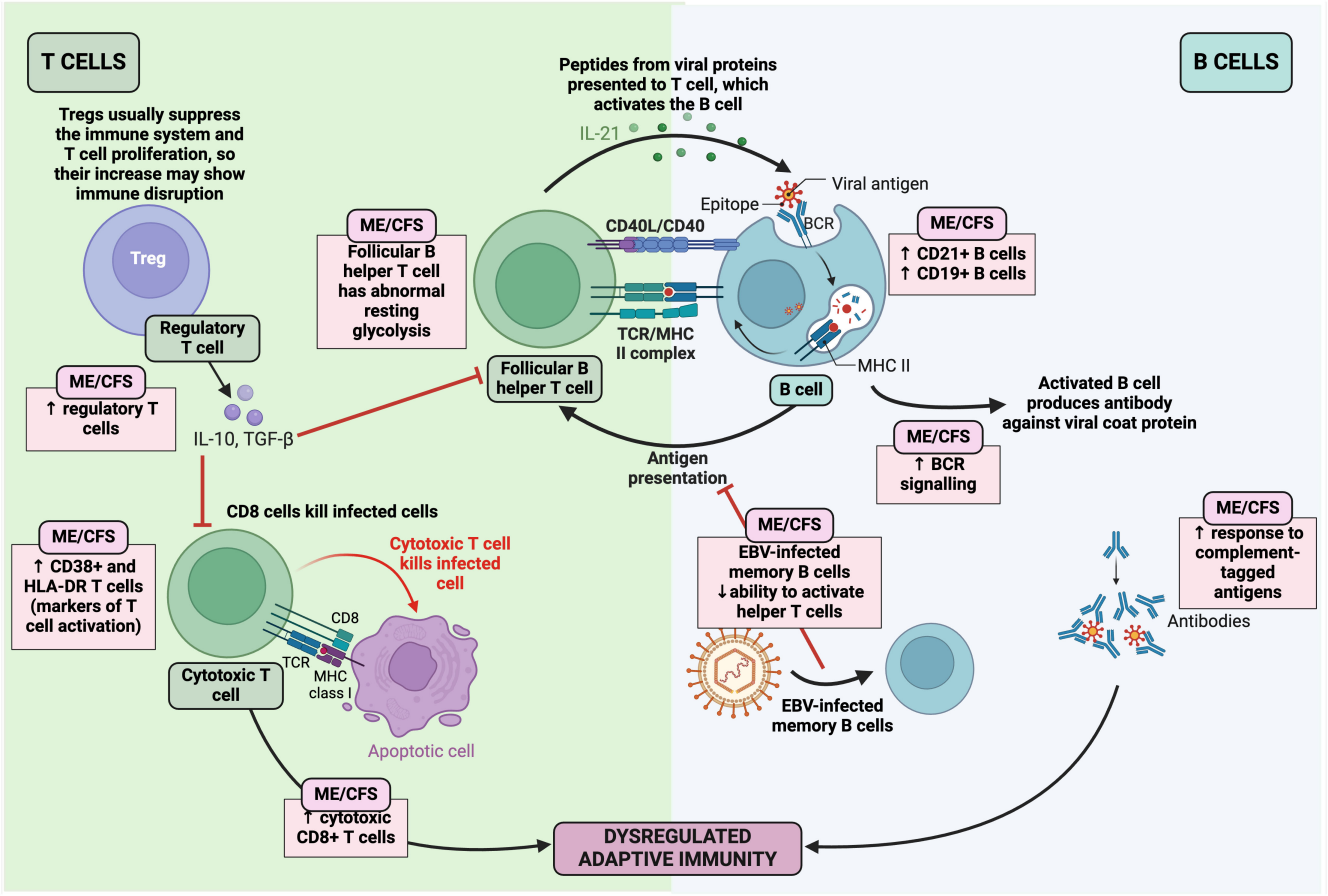
Changes in the adaptive immune system in ME/CFS (25, 52, 201, 278, 445–461, 465–467, 469, 483–485). Created with Biorender.com. BCR, B cell receptor; EBV, Epstein-Barr virus; HLA-DR, human leukocyte antigen- DR isotype; IL, interleukin; ME/CFS, Myalgic Encephalomyelitis/Chronic Fatigue Syndrome; MHC, major histocompatibility complex; TCR, T cell receptor; TGF-β, transforming growth factor-β; Treg, regulatory T cell.

Since one of the most supported hypotheses for the origin of ME/CFS is bacterial or viral (85, 99, 444) infection (94); immune dysregulation has been linked to ME/CFS patients (201, 255) (**Table 2**) (**Figure 11**), it is possible that infectious organisms result in chronic symptoms by interfering with host gene expression, immunity, and metabolism (25). More evidence of an infectious aetiology can be seen in the alteration of the number and function of various immune cells, immune profiles, and autoimmune parameters (294, 486). It has been noted that these abnormal immune responses may be more pronounced within the first three years of the disease; as the disease becomes prolonged, these abnormalities appear to subside and T-cell exhaustion becomes evident (225, 254). This suggests that the overactive immune response eventually becomes exhausted or overcome by counter-regulatory mechanisms. Since a link between infections and autoimmune diseases has been well established, this correlation could explain the presence of autoimmune symptoms in ME/CFS (487). However, the measurement of the innate and adaptive immune responses in ME/CFS patients has resulted in conflicting abnormal results (235). Although immune dysfunction is clear in ME/CFS, further studies using defined cohorts, standardised assays, and new technologies are required to determine specific patterns (30). Furthermore, the use of antiviral drugs to treat ME/CFS has been unsuccessful (488), contradicting the hypothesis that ME/CFS could be caused by a clearance failure of the pathogenic microbe (99, 107). A further hypothesis is that ME/CFS could include misdirected immune responses to the initial infection, resulting in a chronic autoimmune disease with molecular mimicry (the “hit and run” hypothesis) (85).

**Table 2:** Immune alterations present in ME/CFS and changes evident in B cells, T cells, and NK cells (99, 253–255, 278, 445–482).

Cell type	Abnormalities	Comments
B cells	Increase in B cells (445), including CD21+, CD19+, and CD5+ B cells (446, 447), and antigen-driven clonal B cell expansion (448).	CD19/CD21 complex: Promotes BCR signalling in response to complement-tagged antigens (449). CD5+ B cells: Likely involved in antigen presentation, tolerance induction, the idiotype network, and autoantibody production (450).
Cytotoxic T lymphocytes (CD8)	Increase in activated CD8+ T cells (451–453) expressing activation markers HLA-DR (446, 451, 452, 454), CD26 (446), and CD38+ (451, 452, 454). A reduction in CD11b levels was observed (454). Some papers have illustrated a reduced response of T cells to mitogens and antigens (253–255). Also found decreased CD8 suppressor cell population (452).	CD38: T cell surface protein that contributes to cell activation (455, 456). HLA-DR: A marker of T cell activation (457, 458) that is also increased in autoimmune diseases (459).
	Decreased cytotoxicity of CD8+ T cells (253, 460, 461, 483).	It is possible that these changes reflect T-cell exhaustion in prolonged disease duration (462).
	At rest and upon activation, the CD8+ T cells had a reduced mitochondrial membrane potential (254). Additionally, one subset of CD8+ cells had an elevated mitochondrial mass (254).	Reduced mitochondrial membrane potential: This phenotype may be indicative of T cell exhaustion and is typically observed in chronic viral infection (463). Increased mitochondrial mass: Suggests impaired mitochondrial and glycolytic metabolism in ME/CFS T cell subsets (464).
Regulatory T cells (Tregs)	Increased Treg cells in ME/CFS patients (278, 465–467). However, some studies did not take the stages of the illness into consideration [much as in ostensibly conflicting accounts of acute COVID-19 (468)], nor were they sufficiently powered to stratify subtypes. As a result, there is inconclusive literature around whether Tregs are increased or decreased in ME/CFS (451, 467).	Tregs work to suppress the immune system by inhibiting T cell proliferation and cytokine production, while also preventing autoimmunity (469). Hence, an increase in Tregs may show disruption of the immune system since this subpopulation functions to suppress the immune response.
T-follicular helper cells (CD4)	The resting glycolysis of the ME/CFS CD4+ T cells at rest was found to be significantly lower than cells from healthy controls (254).	In cases of short-term, rapid fuel production, remodelling of CD4+ T cells is possible to elevate glycolysis and optimise oxidative phosphorylation (464).
Natural killer (NK) cells	NK cell functioning is reduced in ME/CFS patients (470–473). Conversely, an elevation in CD16+/CD3- NK cells has been found in some ME/CFS patients (452).	NK cells form part of the innate immune system and control various microbial infections by preventing their spread and subsequent tissue damage (475). Hence, this beneficial functioning is reduced in ME/CFS.
	Reduction in NK cytotoxicity (446, 451, 476, 477). Conversely, some studies have not found a decreased cytotoxic activity of NK cells (99, 478–482).	

BCR, B cell receptor; CD4, T-follicular helper cell; CD8, cytotoxic T lymphocyte; HLA, human leukocyte antigen; ME/CFS, Myalgic Encephalomyelitis/Chronic Fatigue Syndrome; NK, natural killer cell; Treg, regulatory T cell.

## Autoimmunity

### Upregulation of autoimmune genes

As in other chronic disorders, some genes in ME/CFS patients have been found to be associated with autoimmunity, such as the HLA alleles (30). In ME/CFS, an increased prevalence of the class II major histocompatibility complex HLA-DQB*01 allele (88), along with two other variants of HLA-DQB1 in combination with two RAGE-374A variants (489) was found. Although some authors have tried to assess the mechanistic link between HLA-II allele expression and the development of ME/CFS, the lack of robust data makes it difficult to support this association (88, 490). Single nucleotide polymorphisms (SNPs) in receptors, enzymes, and transcription factors are also known to cause loss or gain of functions (30) that can increase the risk of autoimmune disease development (491–495). If such changes occur in T cell development, B cell activation and proliferation, or cytokine signalling, it may result in the development of autoimmune diseases. In ME/CFS, SNPs in TLR signalling pathways, the complement cascade, and cytokines have been identified (74, 496).

As mentioned, infectious mononucleosis caused by EBV is a risk factor for various autoimmune diseases (487, 497), and may play a potential role in ME/CFS (140, 498–500). After being infected by EBV, some ME/CFS patients have upregulation of the EBV-induced gene 2– an important gene in immune and CNS function (25). Hence, such gene induction may correlate with various neurological and immune-related symptoms of ME/CFS, as an estimated 38-55% of ME/CFS patients have symptoms that overlap with other autoimmune diseases (501). Similarly, one study illustrated an enhanced IgG reactivity against an EBV repeat sequence, EBNA-6, suggesting that homologous sequences of various human proteins with this repeat sequence might be useful targets for antigenic mimicry (85).

### Autoantibody presence

It has been proposed that ME/CFS is a variant of an autoimmune mechanism (29). Typical autoimmune diseases have characteristic pathogenic IgGs correlated with inflammation, tissue injury, and complement activation. Such persistent autoantibodies are hypothesised to disturb vessel autoregulation, causing secondary metabolic and autonomic adaptations. Although these typical changes are not necessarily characteristic of ME/CFS, another autoimmune mechanism may exist in ME/CFS that affects the autonomic control of blood vessel tone and flow autoregulation. Hence, autoimmunity is considered important in the pathology of ME/CFS (30, 85, 182).

In some ME/CFS patients, autoantibodies have been identified, including those against antinuclear antibodies (502–505). These autoantibodies are hypothesised to target nuclear, membrane, and neurotransmitter receptor structures (30, 255). Double-stranded DNA antibodies have also been found in 12% of ME/CFS patients (506), although other studies have failed to find such antibodies in ME/CFS (0.7%) (507). Single-stranded DNA antibodies have also been identified (505), along with anti-ganglioside antibodies (508), autoantibodies against endothelial and neuronal cells (506), and phospholipid autoantibodies (506, 508, 509). Antibodies against cardiolipin were also found in 92-95% of ME/CFS patients (509, 510), although they were only 4% in another study (506). Additionally, antibodies have been identified against human nuclear dUTPase and nuclear envelope protein lamin B1 (511). Hence, this autoantibody presence especially targets the autonomic and central nervous systems (503), which may explain the dysautonomia and immune dysregulation present in ME/CFS (58).

In other autoimmune diseases, natural antibodies are found against adrenergic, muscarinergic, and other G protein coupled receptors (GPCR) (30, 512). In some ME/CFS patients, such antibodies against neurotransmitter receptors have been identified, such as against the muscarinic M1AChR (504), M3AChR (58, 359, 503), and the adrenergic B2AdR (58, 359, 503). The presence of these autoantibodies potentially results in dysfunction in these receptors (58, 513). However, no difference between ME/CFS patients and controls was found with respect to autoantibodies against serotonin, angiotensin, endothelin, mu-opioid, and dopamine (503, 504). Although, autoantibodies against serotonin have been associated with ME/CFS (506, 508).

Various general autoantibodies have also been identified in ME/CFS, such as those against cellular components including anchorage molecules (514), HSP-60 (515), microtubule associated protein 2 (516), cardiolipin in 92-95% of ME/CFS patients in two studies (509, 510) but only 4% in another study (506), and neo-antigens (517). Moreover, 30% of ME/CFS patients in one study were identified to have antibodies against endothelial cells (506). If autoimmunity is present in ME/CFS, it may increase intestinal permeability (518) and explain the various GI manifestations (190).

### Soluble autoimmunity markers

B lymphocyte activating factor (BAFF) has been identified in many autoimmune diseases (519) as it regulates survival and maturation of B cells to control the IL-10 production of regulatory B cells (520, 521). Some ME/CFS patients have displayed elevated BAFF, but the gene expression of the BAFF receptor (TNFRSF13C) has been shown to be reduced in ME/CFS patients, suggesting that the elevated serum BAFF is a compensatory mechanism. However, the link between BAFF and autoantibodies in ME/CFS is yet to be investigated (30). Members of the TGF-β family- Activin A and B- are known to control inflammation and muscle mass (522). Heightened levels of activin A and B have been found in ME/CFS with an elevated ratio of activin A or B to the binding protein follostatin (523). Although no causal role has been established, activin A is a pleiotropic cytokine known to influence immune regulation and is altered in various autoimmune and inflammatory diseases (524). IL-21 is also a pleiotropic cytokine (525) important for differentiation of follicular helper T cells that are essential for the germinal centre antibody response (526). When activin A (527) and IL-21’s (528) processes are dysregulated, it is hypothesised to promote autoimmune and inflammatory diseases, although a causal role is still not shown (527).

## Management of ME/CFS

**Figure 12:**
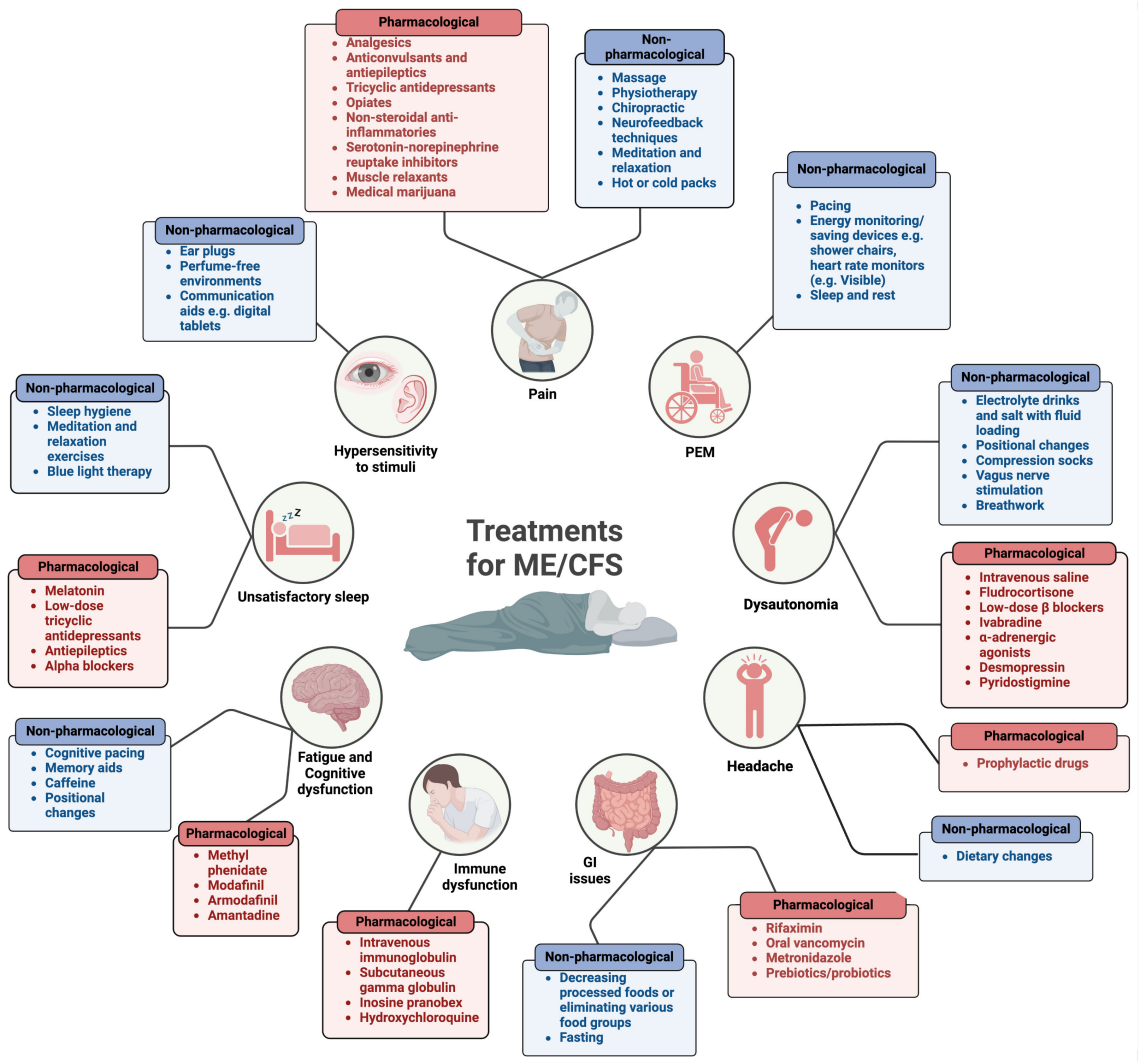
Pharmacological and non-pharmacological treatments currently available for ME/CFS (11, 23, 60, 532). Created with Biorender.com. GI, gastrointestinal; PEM, post-exertional malaise.

As well as there being insufficient diagnostic testing available for ME/CFS patients, there are also no effective therapies (12, 26, 60, 488, 529–531) and few established non-pharmacological treatments for ME/CFS (11, 29). The lack of awareness (60), paucity of diagnostic tools (60), heterogeneity between patients (26, 60, 529), disbelief from health care workers (60), unpredictable relapses, and multiplicity of symptoms have made it difficult to formulate a treatment for ME/CFS. Hence current advice is aimed at symptom management and lifestyle changes (11). The current available treatments/lifestyle modifications are summarised in **Figure 12**.

### Disbelief of doctors

Since not enough is known about the risk factors of ME/CFS, it makes primary prevention unlikely (53). However, secondary prevention is possible to reduce diagnostic delay, the incidence of severe and prolonged disease, and costs of care. One way to minimise diagnostic delays is to rectify the disbelief of doctors and the stigmatization around ME/CFS (53, 533, 534). In 1969, the WHO classified ME/CFS as a neurological disease (44) based on the neurological features of the disease. Following this, epidemic cases of ME/CFS were described as “mass hysteria” in 1970 by two psychiatrists, McEvedy and Beard (535). Although these psychiatrists were faulted for inadequately investigating the patients they described (536) and their conclusions were discredited (537–539), this sparked stigmatization of ME/CFS.

Another mistake that doctors sometimes make is to second-guess their initial diagnosis (39). This scenario may arise when doctors experience uncertainty or apprehension about their assessment, potentially leading to ME/CFS patients feeling neglected. This uncertainty or hesitation may prompt doctors to seek confirmation from other professionals, which may create a traumatic experience for the patient and prolong their diagnosis. Although, since ME/CFS is currently diagnosed based on exclusion criteria and subjective symptom assessment, it is sometimes necessary for patients to be referred to other professionals to exclude other possible diagnoses. For example, referral to psychiatrists may be helpful since ME/CFS seems to coexist with anxiety disorders, symptoms of ME/CFS overlap with Major Depressive Disorder (MDD), and patients with severe ME/CFS may also be at risk of developing secondary MDD (36, 532). However, it can also be detrimental referring ME/CFS patients to psychiatrists (39), not because it excludes other diagnoses, but rather if patients are labelled as hypochondriacs and their condition is attributed solely to psychosomatic origins (23). Hence, validation of the patient’s experiences is important. Additionally, friends and family must offer support to patients, such as helping them acquire handicap placards, attain work and school accommodations, make nutritional adjustments, and apply for disability and housing benefits (23).

To manage and live with ME/CFS, it is also important that patients schedule regular trips to their physician to manage their symptoms, as well as discuss complementary approaches the patient may have adopted- such as new supplements (23). This communication is important to minimise any adverse side effects or drug interactions that may occur (540). Additionally, the reassurance of doctors is important, and they should openly address questions related to a patient’s prognosis. This will help patients maximise their functioning and enhance their quality of life (23). It is worth pointing out that more severe patients are likely to tolerate visits to their doctor poorly, and home visits should be undertaken for them.

### Pacing/energy management

Several guidelines have been established to guide the management of ME/CFS. However, some of the recommendations have possibly resulted in more harm than good. One controversial form of self-help is physical activity. When ME/CFS was still believed by many to be a psychological disease (23), doctors often prescribed inappropriate “treatments” such as cognitive behavior therapy (CBT) (39, 530), graded exercise therapy (GET) (39, 530), or the Lightning Process (11) to ME/CFS patients. In 2007 for example, the National Institute for Health and Care Excellence (NICE) released a guideline for clinicians and patients where GET was recommended as a treatment (541). GET first establishes a patient’s baseline of physical activity, and this is then stepped up in fixed increments (11). However, these “treatment” strategies have now been strongly criticised for the harm they have caused (542–544), and in October 2021, GET was removed from the revised NICE guidelines (11). Although, it is important to note that while CBT may not be appropriate as a cure for ME/CFS itself, it could be applied in ME/CFS patients to address symptoms or secondary disorders such as MDD (545).

Furthermore, one misleading and now entirely discredited study (546) was the 2011 PACE (Pacing, Graded Activity, and Cognitive Behavior therapy; a Randomised Evaluation) trial (547). In this paper, the therapies were described as safe with 22% of participants recovering and 60-61% of patients experiencing symptom improvement (547, 548). However, there were specific flaws identified in this paper. Without any clear rationale, the study outcome measures were purposefully modified midtrial to alter the findings in favour of their hypothesis (549, 550). Additionally, patients could worsen during the study and still be classified as “recovered” and the study was unblinded with subjective outcomes. Subsequent review of the raw data revealed that the improvement and recovery rates were not significantly different from the control participants. Moreover, 54% to 74% of patients revealed that they experienced harm after GET (543).

Instead, energy management (pacing) is now emphasised for symptom control (11). Pacing is an individualised approach that monitors energy expenditure to reduce the occurrence, duration, and severity of PEM (23). Energy expenditure encompasses physical, social, cognitive, and emotional activity (11). Avoidance of PEM can help decrease fatigue, cognitive difficulties, sleep disruption, and other symptoms of ME/CFS (551). Some patients use energy monitoring and saving devices such as shower chairs and heart rate monitors (552). Although many commercially available trackers such as Fitbit and Garmin encourage exercise and exertion, the Visible application is a recently designed activity monitor for those with chronic illness (553). According to the amended NICE guidelines, an individualised approach is more appropriate for ME/CFS patients (11). If an ME/CFS patient wishes to incorporate physical activity, it should be closely monitored by a team of specialists as over-exertion has the potential to worsen the symptoms of ME/CFS. Hence, it is the patient’s responsibility to gauge their activity levels while a physiotherapist or occupational therapist oversees the activity. Additionally, patients should be wary of potential relapses or flare-ups to prevent worsening of symptoms.

### Nutraceuticals and pharmacological approaches

Many ME/CFS patients take nutritional supplements (530). Vitamin D supplementation can help prevent a vitamin D deficiency (11, 39). If bedbound for extended periods of time, bisphosphonates can also help reduce the risk of osteoporosis (39). If toxin exposure is thought to have triggered the development of ME/CFS, nutritional supplementation of zinc and magnesium may be beneficial in the prophylaxis and therapy of cadmium exposure (154). More research is required around nutraceuticals to optimise their dosages.

Despite there being no U.S. Food and Drug Administration (FDA)-approved treatments for ME/CFS (40), in many cases the symptoms and comorbidities experienced by patients can be treated (24). However, it is important that any medication should be introduced at low dosages as many ME/CFS patients are extremely drug sensitive (23), making them vulnerable to adverse side effects. Since ME/CFS was psychologised at first, many patients were initially treated with antidepressants (60). This misdiagnosis resulted in adverse side effects and dependence on the drug. However, antidepressants are still sometimes prescribed to help if patients are experiencing depression (39).

### Treating comorbidities

ME/CFS displays an overlap with several autoimmune or immune-mediated diseases that also have chronic fatigue as a main symptom, such as Hashimoto’s thyroiditis (530), fibromyalgia (FM) (532, 554–557), Mast Cell Activation Disorder (532), sleep apnoea (532), IBS (532), secondary depression or anxiety (532), Ehlers-Danlos syndrome (532), and PoTS (532, 558–563). One ME/CFS speciality clinic found 84% of 960 patients presented with at least one other comorbid condition, often resulting in worsened health (3). Hence, more research on ME/CFS is required to separate it from other diseases that are also associated with chronic fatigue. Treating some of these comorbidities will not cure ME/CFS but might alleviate some of their symptoms and improve some quality of life (23).

### Potential therapies

**Figure 13:**
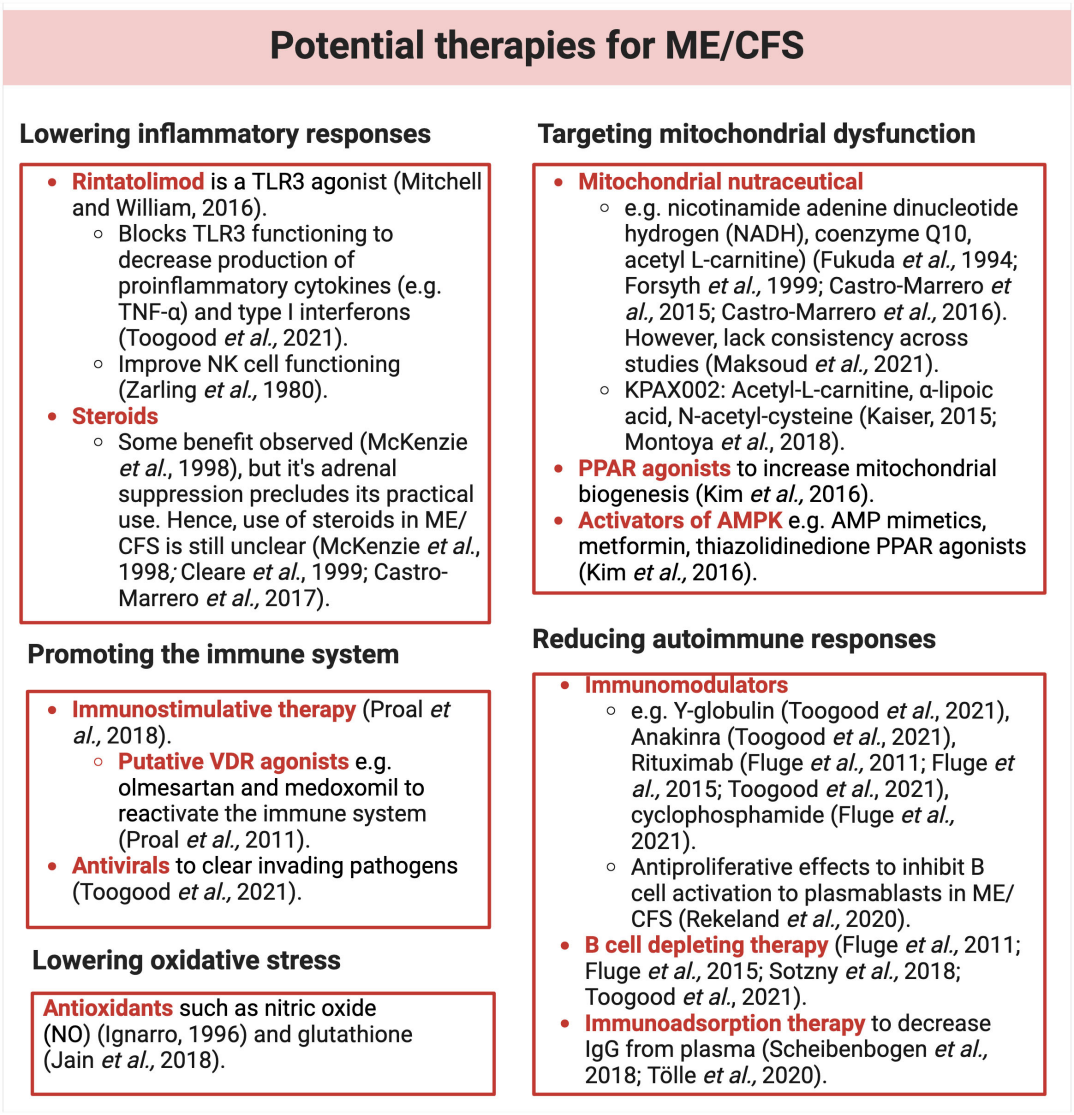
Potential therapies that could be beneficial in the treatment of ME/CFS (22, 29, 29, 30, 84, 193, 530, 564–580). Created with Biorender.com. IgG, immunoglobulin G; ME/CFS, Myalgic Encephalomyelitis/Chronic Fatigue Syndrome; NK, natural killer; NO, nitric oxide; PPAR, peroxisome proliferator-activated receptor; TLR3, toll-like receptor 3; VDR, vitamin D receptor.

Although some researchers have attempted to establish a treatment for ME/CFS and no reproducible results have been found (60), some treatments seem promising in ME/CFS (**Figure 13**). Additionally, not many trials meet the clinical design standards to be considered credible. Although no causation has been established for ME/CFS, as the knowledge around the disease expands, so will the potential treatment avenues. Hence, pathways and mechanisms need to be investigated for possible pharmacological intervention. Considering the heterogeneity among ME/CFS patients, however, it is unlikely that one treatment will suit all ME/CFS patients.

## Future research recommendations

Most of the research and clinical studies conducted on ME/CFS have inconsistent outcomes and use different study parameters, making the results difficult to compare or combine (11). Most studies are limited owing to their insufficient sample sises, short follow-up times, high dropout rates, inappropriate definitions of recovery, and inclusion of patients with varying conditions (581, 582). Additionally, the small cohort in many studies along with limited comparison groups with other fatiguing illnesses cause inconsistent findings (201). To improve their reproducibility, studies need to use uniform clinical and research criteria, standardise sample collection, and use thorough statistical analyses (305).

Although the research surrounding ME/CFS has increased in the last decade (29), there is still a significant lack of funding for research surrounding ME/CFS (60). In the U.S., the National Institutes of Health (NIH) receives an overall budget of 42 billion dollars. However, in 2019, they only budgeted 15 million dollars towards ME/CFS research, whereas 111 million dollars and 94 million dollars was donated to MS and RA research, respectively (both of which already have drugs on the market). More research is required to define and diagnose ME/CFS as a serious chronic illness (25), as it is uncertain how many patients diagnosed with ME/CFS have an accurate diagnosis (57). Not only does the diagnosis of ME/CFS need to be more accurate, but it also needs to be timely to ensure an earlier diagnosis before a prolonged disease progression occurs (11). Standardisation in diagnosis and disease severity is required to create robust and reproducible results. In combination, more effort needs to be made to include correctly matched controls. For example, since age, lifestyle, medication, geography, and diet are all able to impact microbiome function and composition (583–585), detailed descriptions of food consumption at least 48 hours before the sample collection should be included. Moreover, no defined clinical criteria for ME/CFS makes it even more complicated to compare various study results (52). Additionally, a large GWAS in ME/CFS is desperately needed to assess the biomolecular mechanisms of ME/CFS (56). One study aiming to achieve this is the DecodeME study that launched September 2022 (586). This study aims to pinpoint genetic causes of ME/CFS by testing more than 25,000 DNA samples (586), as a large cohort will help achieve statistical power with reproducible and rigorous results (52). Similarly, identifying microbiomes in ME/CFS blood and brain tissue should be prioritised in ME/CFS research (193). Since some of the main components of ME/CFS include redox imbalance, chronic inflammation, and defective energy metabolism, it is also important to investigate how these components interact bidirectionally (201).

One thing to consider is that because ME/CFS is a severely disabling disease, many patients are house or bedbound and are unable to visit the clinics multiple times for follow-up analyses (39), even though these very severe cases are estimated to be around 25% of the ME/CFS cases (11, 39, 587). It is important to make research more available for this percentage of ME/CFS cases, since they are the most severely affected by the disease and these severe cases are rarely studied (10). It is likely that as the condition worsens, so the probability of identifiable biomarkers for the disease increases, making it crucial to study severely ill patients. ME/CFS needs to be more patient-centered, involving patients in clinical decisions regarding their treatment (11, 53). There is also concern that the COVID-19 pandemic may increase the number of people suffering from ME/CFS (588). Similarly, with the growing emergence of Long COVID cases, many Long COVID symptoms are coinciding with those present in ME/CFS (29). More research is required to determine whether these symptoms are caused by subtle organ damage from the viral infection, or whether these Long COVID patients have a postinfectious immune disturbance whose pathomechanisms is like that in ME/CFS.

It is also important to break the negative stigma that is associated with ME/CFS. ME/CFS is a remarkably misunderstood disease, and many patients have experienced judgement, prejudice, and disbelief from healthcare professionals (11, 39) as some refuse to view ME/CFS as an indisputable clinical entity (39) and question its legitimacy (25). This disbelief and misjudgment (40, 58) delays any possible early diagnosis for the patients and has also resulted in psychologisation of the disease (11, 40, 53). Health and social care professionals need to acknowledge that their patients are living with ME/CFS, and how they have symptoms that severely affect them (11). Hence, they should take time to build empathetic relationships with their patients.

Finally, it is imperative to provide increased support to the ME/CFS community, a necessity that is steadily growing alongside the establishment of research groups and charitable organisations dedicated to aiding ME/CFS patients. One multidisciplinary team consisting of ME/CFS researchers and health professionals, the European Network on ME/CFS (EUROMENE), aims to evaluate healthcare for ME/CFS in Europe, improve research and services in the field, and grasp socioeconomic and clinical dimensions of the disease to issue recommendations accordingly (589). The Open Medicine Foundation is another collaborative effort dedicated to advancing medical research on ME/CFS, fostering engagement within the patient community, and promoting education about the condition (590). The Charité Fatigue Centre in Berlin not only conducts clinical research, but also offers support to both patients and doctors in diagnosing and treating ME/CFS, making it an invaluable interdisciplinary network (591). Additionally, registered charities such as the Austrian “WE&ME” Foundation are dedicated to financing ME/CFS research endeavors and raising awareness about the condition (592). Similarly, “Action for M.E.” in England and Wales aim to spread awareness and education around ME/CFS and raise funds to support these patients and their families. They have also recently worked together with the James Lind Alliance non-profit organisation, ME/CFS clinicians, and ME/CFS sufferers and caretakers to identify ME/CFS research priorities (593). The top three research priorities were found to be (1) understanding the biological mechanism behind PEM and how this can be treated or managed (2), which existing drugs used for other conditions would be useful in treating ME/CFS, and (3) finding accurate and reliable diagnostic testing for ME/CFS. Hence, future research should include recommendations such as these with personal input from ME/CFS sufferers.

## Author contributions

EP: Conceptualization, Funding acquisition, Supervision, Writing – review & editing, Resources, Validation. HA: Project administration, Validation, Visualization, Writing – original draft. MK: Writing – review & editing. BJ: Writing – review & editing. BM: Writing – review & editing. DK: Writing – review & editing.
